# Next-Generation Hydrogel Platforms for Effective Localized Cancer Therapy: Advances in Biologics, Immunotherapeutics, and Gene Delivery

**DOI:** 10.32604/or.2026.074061

**Published:** 2026-03-23

**Authors:** Vincenzo Montanarella, Marcelo Guerrero, David Filho, Júlia German-Cortés, Giacomoluciano Vitelli, Magalí Sureda, Carlos Pavón Regaña, Roser Ferrer, Simó Schwartz, Esteban Durán-Lara, Fernanda Andrade, Diana Rafael

**Affiliations:** 1 Clinical Biochemistry, Drug Delivery and Therapy Group (CB-DDT), Vall d’Hebron Institut of Research (VHIR), Vall d’Hebron University Hospital, Vall d’Hebron Barcelona Hospital Campus, Passeig de la Vall d’Hebron, 119-129, Barcelona, 08035, Spain; 2 Bio & Nano Materials Lab, Drug Delivery and Controlled Release, Departamento de Microbiología, Facultad de Ciencias de la Salud, Universidad de Talca, Talca, 3460000, Chile; 3 Center for Nanomedicine, Diagnostic & Drug Development (ND3), Universidad de Talca, Talca, 3460000, Chile; 4 Clinical Biochemistry Service, Vall d’Hebron University Hospital, Vall d’Hebron Barcelona Hospital, Barcelona, 08035, Spain; 5 Department of Pharmacy and Pharmaceutical Technology and Physicochemistry, Faculty of Pharmacy and Food Sciences, School of Pharmacy, Universitat de Barcelona (UB), Av. de Joan XXIII, 27-31, Barcelona, 08028, Spain; 6 Functional Validation & Preclinical Research (FVPR)/U20 ICTS Nanbiosis, Vall d’Hebron Institut de Recerca (VHIR), Barcelona, 08035, Spain

**Keywords:** Cancer treatment, hydrogels, biomolecules, peptides, immunotherapy, gene therapy, local therapy, sustained release

## Abstract

Despite remarkable advances in nanomedicine, localized delivery of advanced cancer therapeutics remains underexploited. Advanced therapies based on biopharmaceuticals, immunotherapy, or gene therapy have revolutionized oncology. Yet, their systemic administration is often associated with limitations such as poor site-specific accumulation, instability, and systemic toxicity. Hydrogels/macrogels offer the ability to encapsulate, protect, and release biomolecules *in situ* with sustained and stimulus-responsive profiles, addressing key translational gaps. This review provides a focused synthesis of the last five years of hydrogel-based research for cancer therapy, with emphasis on peptides, antibodies, immunotherapeutic agents, and gene delivery systems. We discuss design principles, release mechanisms, and clinical translation challenges, highlighting structure–function relationships and comparative performance across therapeutic classes. By integrating mechanistic insights with recent breakthroughs, we outline how next-generation hydrogels can synergize with personalized medicine and combination therapies to redefine localized cancer treatment. This work explores the fundamental aspects and provides examples of hydrogel-based delivery for the advanced treatment of cancer. The review summarizes the dynamic landscape of hydrogel research of the last 5 years, showcasing their potential systems for the precise delivery of biomolecules. Specifically, we explore the multidimensional role of hydrogels in the sustained and localized release of antibodies, immunotherapeutic agents, and genes as next-generation platforms for localized cancer treatment. This review aims to critically evaluate the mechanisms and applications of these systems in order to assess their potential to transform medical interventions and advance patient care.

## 1 Introduction

In the realm of modern medicine and nanotechnology, the sustained and localized delivery of biomolecules ranging from pharmaceuticals to nucleic acids holds immense promise for revolutionizing therapeutic interventions [1]. Over the past decades, nanotechnology-based delivery systems have significantly contributed to cancer therapy, as reflected by the growing number of approved nanomedicines [2]. Also, it holds the potential for biomolecules and advanced therapies. However, they still suffer from challenges such as limited control over release, suboptimal targeting, and the need for frequent systemic administration [3]. Local delivery appears as an alternative to systemic therapies, not only because it increases the concentration of the therapeutic compounds in the site of action but also reduces the systemic side effects [4]. However, the fine balance between efficient delivery, sustained release, and preservation of biomolecular integrity presents a significant challenge [5]. In this sense, hydrogels have emerged as versatile and dynamic carriers that offer ingenious solutions to this challenge [6]. These three-dimensional polymeric networks, characterized by high water-absorption and retention capacity, provide a customizable platform for local therapeutic delivery and are poised to reshape the landscape of biomolecule delivery [7]. Their biocompatibility, tunable physical properties, and potential for responding to different stimuli based on the polymers used in the composition make them especially well-suited for modern drug-delivery applications [8]. Moreover, hydrogels’ aqueous and hydrated environment closely mimics the physiological milieu, promoting biomolecule stability and bioactivity [9].

Hydrogels can be categorized according to several characteristics, such as size (nanogels, macrogels, etc.), composition, crosslinking, etc. Their design and fabrication depend on the properties of the formulation components and the intended application, and are generally divided into chemical and physical approaches [10]. Chemically crosslinked hydrogels are produced through the formation of covalent bonds between polymer chains, resulting in networks with enhanced mechanical stability and highly tunable characteristics [10]. This can be obtained by grafting monomers to the macromolecular backbone or using agents that link two polymer chains. Typical strategies include chemical reactions such as aldehyde-mediated crosslinking (e.g., glutaraldehyde), carbodiimide-mediated condensation, and divinyl sulfone addition, as well as radical-free polymerization (e.g., UV light), high-energy radiation crosslinking (e.g., gamma rays and electron beam), or enzymatic crosslinking [11,12]. In contrast, physically crosslinked hydrogels are formed by molecular entanglements, and/or secondary forces including ionic interactions, hydrogen bonding, hydrophobic interactions, crystallization, protein interactions, metal coordination, or nanoparticle-driven self-assembly [11,12]. These systems typically exhibit lower mechanical strength, quicker degradation, and lower residence time than chemically crosslinked hydrogels. However, they offer notable advantages: they are easier to produce since they require fewer synthetic steps, are often more biodegradable, and avoid the toxicity associated with some chemical crosslinkers. Additionally, their non-covalent nature provides reversibility, allowing structural disruption in response to environmental changes or applied mechanical stress [10].

**Figure 1:**
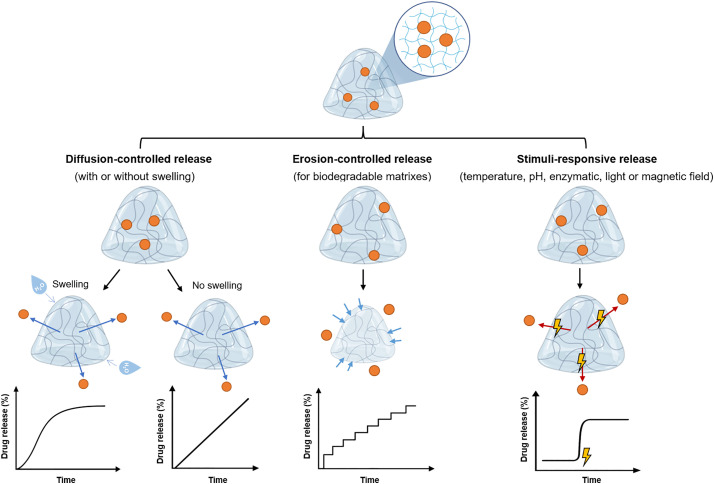
Schematic representation of the different release mechanisms of therapeutic compounds from hydrogels. Created with Biorender.com

Drug release from hydrogels can occur through diffusion-controlled mechanisms, driven by the drug concentration gradient and often enhanced by polymer network swelling [13,14], through polymer erosion or degradation, which governs release in biodegradable systems [15], or through stimuli-responsive mechanisms, in which external or physiological triggers such as pH, temperature, enzymes, or redox conditions modulate the network structure and thereby the release profile [16] (Fig. 1). It is noteworthy that the design of hydrogel-based biomolecule delivery systems involves careful consideration of factors such as polymer nature, crosslinker nature, polymer concentration, type and density of crosslinking, porosity, release kinetics, and biocompatibility [17]. For example, alginate-based hydrogels crosslinked by disulfide-tetrazine or disulfide-maleimide present different porosities, gelation times, and drug release rates [18]. During the alginate-disulfide-tetrazine conjugation (inverse electron demand Diels–Alder reaction), nitrogen gas is produced, forming a porous gel. On the other hand, a non-porous formulation is obtained using disulfide-maleimide as a crosslinker through the Diels-Alder reaction. Moreover, by varying the polymer:crosslinker ratio, it is possible to tune some properties, namely the gelation time and porosity. Higher crosslinker density leads to lower gelation time and a small porous size but higher surface area. Higher porosity leads to a faster release of the drug [18]. Diverse types of hydrogels present a rich landscape for biomolecule delivery applications, each catering to specific requirements and challenges [19]. Among them, the thermo-, photo-, and pH-responsive hydrogels have been highly demanded for precision in the delivery [7,20]. The ability of these hydrogels to undergo a transition from a hydrated, water-swollen state to a more compact gel-like state in response to temperature, light, or pH variations introduces a novel mechanism for the sustained and localized release of encapsulated biomolecules [7,20]. The synergy between sustained and localized release capabilities of hydrogels presents a powerful approach to biomolecule delivery. By integrating both mechanisms, hydrogels can provide a constant supply of therapeutic agents while ensuring their delivery exclusively to the intended site [21]. This strategy is especially beneficial in cancer treatment, where sustained release maintains therapeutic levels over time, while localized release minimizes unnecessary exposure to healthy tissues [4]. Based on this, in recent years, many hydrogel-based formulations have been proposed for cancer treatment and extensively reviewed elsewhere [7,20,22–24]. However, to our knowledge, few works are focused on the use of hydrogels for the local delivery of peptides, biomolecules, and immunotherapy intended to treat cancer. In the sections that follow, we explore recent studies from the past five years on hydrogels, focusing on macrogels, for local delivery, with the aim of highlighting their transformative potential for delivering peptides, antibodies, immunotherapeutic agents, and gene therapies.

## 2 Hydrogels for Local Delivery of Peptides

Peptides are short amino acid sequences (10 to 100 amino acid units) that have considerable applications in the biomedical field, mainly due to their variety of properties, including antibiotic and anticancer activity [25]. Their small size, high tumor penetration, specificity, and fewer side effects make them potential alternatives to anticancer drugs [26]. However, enzymatic degradation and instability make its use difficult as systemic chemotherapy [27]. One way to solve these problems is the incorporation of anticancer peptides in hydrogels for their protection and localized and sustained release. Some peptides, in addition to having biological activity, can be used as a component of the hydrogel since the molecular characteristics of the peptides also allow them to create a 3D network of peptidic fibers and respond to stimuli such as temperature, redox environment, and pH [28].

Peptides and their controlled release from hydrogels for local cancer therapy have been studied and proposed by different research groups with promising results. Resina et al. designed an electrochemoreactive carrier for the controlled release of a highly hydrophilic anticancer peptide, CR(NMe)EKA, using electrosensitive poly(3,4-ethylenedioxythiophene) nanoparticles. This system allows for the on-demand release of peptides through electrical stimuli, and encapsulation within a pH-sensitive hydrogel composed of chitosan-grafted phenylboronic acid improves its stability in acidic tumor environments. The released peptide retains its bioactivity and effectively promotes the death of human prostate cancer cells [29].

Stimuli-responsive hydrogels offer extensive design versatility, enabling diverse functional combinations. For instance, enzyme-sensitive hydrogels can undergo controlled degradation in response to specific enzymatic activity, thereby facilitating the sustained release of anticancer peptides as demonstrated by Chen et al. Specifically, short amphiphilic peptides Ac-I3SLKG-NH2 and Ac-I3SLGK-NH_2_ self-assembled into fibrillar hydrogel, with Ac-I3SLKG-NH2 formulation exhibiting excellent biocompatibility and selective degradation in the presence of the matrix metalloproteinase (MMP)-2 enzyme after 15 days. This enzymatically triggered degradation enabled the controlled, “cell-demanded” release of the anticancer peptide G(IIKK)3I-NH_2_, resulting in significant inhibition of HeLa cell proliferation [30].

Another example of a biocompatible self-assembled hydrogel corresponds to the one developed by Yang et al. This work involves the conjugation of chlorambucil (CRB) and tyroservatide (YSV) with hydrogels based on self-assembled peptides, which results in nanofiber hydrogels with greater stability and cellular uptake, leading to greater antitumor efficacy both *in vitro* and *in vivo* against HepG2 liver cancer cells [31]. Increased antitumor efficacy was also achieved by Liu et al., when they synthesized a supramolecular hydrogel with injectability, photothermal ability, and on-demand drug-releasing properties for localized therapeutic delivery and synergistic photothermal–chemotherapy. This hydrogel, created from an anticancer peptide (KL), Fe^3+^ ions, and protocatechualdehyde through dynamic and reversible interactions, exhibiting intrinsic near-infrared (NIR)absorption properties for photothermal ablation of tumor cells, and remote light control of drug release, was designed for doxorubicin delivery. Both *in vitro* and *in vivo* experiments demonstrated that the injectable hydrogel had a synergistic therapeutic effect on inhibiting tumor growth through combined photothermal-chemotherapy [32].

Furthermore, the design of a novel lipopeptide, C16KTTβAH, incorporating the KTT tripeptide, a sequence derived from the natural procollagen I peptide, and the bioactive carnosine βAH (β-alanine-histidine) dipeptide motif, has demonstrated exceptional rigidity and selective cytotoxicity against MCF-7 breast cancer cells. C16KTTβAH self-assembles into nanotape structures above a critical aggregation concentration in an aqueous solution. In PBS, the nanotapes pack side-by-side into raft-like bundles (due to screening of electrostatic repulsions on the charged cationic residues and C terminus). This cytotoxicity is an intrinsic property of C16KTTβAH and is not related to its self-assembly. Furthermore, hydrogels of this lipopeptide demonstrated slow absorption and release of diagnostic compounds such as Congo red dye [33].

Peptides that have membrane activity also attract great attention, in order to facilitate the penetration of active compounds with low permeability through a direct destruction mechanism of the cell membrane of tumor cells. Feng et al. developed a peptide-based stimulus hydrogel composed of melittin-RADA28 hydrogel (RADA is one of the most widely investigated self-assembling peptide hydrogels) for the delivery of KLA peptide, an impermeable peptide. Incorporating melittin in the hydrogel backbone reduces its toxicity and turns it into a substance capable of enhancing the permeability of cell membranes. This proteinase K-responsive hydrogel improves the permeability of the membrane of tumor cells and demonstrates greater efficiency of administration of the therapeutic peptide KLA. Melittin-RADA28-KLA peptide hydrogel increases the cellular accumulation of KLA and significantly inhibits tumor growth *in vivo* and *in vitro* [34].

In another study, the fabrication of an anticancer hydrogelator based on D/L peptide NapGDFDFDYGYSV (D-YSV) resulted in a stable hydrogel with excellent resistance to protease digestion and superior anticancer efficiency *in vitro* due to better cellular uptake. D-YSV also exhibited good biocompatibility and excellent tumor inhibition ability *in vivo* after tail vein injection [35].

Overall, these studies highlight the potential use of peptides with anticancer activity encapsulated in hydrogels. Hydrogels allow us to solve the disadvantages that peptides have, such as the short half-life, susceptibility to enzymatic degradation, and protection of the peptides. Additionally, it enables improved delivery and efficacy of anticancer therapies through several innovative approaches, including electro-controlled release, stimulus-responsiveness, and improvement of membrane permeability. More examples of hydrogels for local delivery of anticancer peptides are presented in Table 1.

**Table 1:** Examples of hydrogel-based formulations for local cancer therapy under development and preclinical evaluation

Category	Hydrogel	Stimuli-sensitiveness	Cargo	Combined therapy	Model	Application	Ref.
	Phenylboronic acid grafted to chitosan (PBA-CS) with electro-responsive poly(3,4-ethylenedioxythiophene) (PEDOT) nanoparticles	Electric and pH	CR(*NMe*)EKA (Cys-Arg-*N*-methyl-Glu-Lys-Ala) peptide	N/A	PC-3 and PNT-2 cells	Prostate cancer	[29]
	Ac-I3SLKG-NH2	Enzymes	G(IIKK)3I-NH2 (G3) peptide	N/A	HeLa or NIH 3T3 cells	Cancer	[30]
Peptides	Self-assembled chlorambucil conjugated with tyroservatide	N/A	Chlorambucil and tyroservatide	Chemotherapy	MCF-7, and BEL-7402 cells and HepG2 tumor-bearing BALB/c mice	Hepatocellular carcinoma and Breast cancer	[31]
	KL peptide	NIR and pH	Fe^3+^ ions and protocatechualdehyde	Chemo-photodynamic therapy	HepG2 tumor-bearing BALB/c mice	Hepatic cancer	[32]
	C16KTTβAH	N/A	KTT tripeptide	N/A	MCF-7 cells	Breast cancer	[33]
	Melittin-RADA28 (MR)	Enzymes	KLA (KLAKLAKKLAKLAK)	N/A	CT26 tumor-bearing BALB/c mice	Colon carcinoma	[34]
	NapGDFDFDYGYSV (D-YSV)	N/A	N/A	N/A	BEL-7402 tumor-bearing BALB/c mice	Hepatoma	[35]
	Vitamin E-functionalized ‘ABA’ triblock copolymers with carbamate block junction	N/A	Trastuzumab	N/A	BT474 tumor-bearing BALB/c mice	Breast cancer	[72]
Monoclonal Antibodies	Thiolated hyaluronic acid	Redox	Immunoglobulin G	N/A	NA	Cancer	[73]
	Maleimide-modified γ-polyglutamic acid (γ-PGA-MA) and thiol end-functionalized 4-arm poly(ethylene glycol) (4-arm PEG-SH)	N/A	Trastuzumab	N/A	BT474 tumor-bearing NOD/SCID mice	Breast cancer	[74]
	Pluronic^®^ F127	Thermal	CTLA-4 checkpoint blocking antibodies	N/A	CT26 tumor-bearing BALB/c mice	Colon cancer	[75]
	Pluronic^®^ F127-polyethyleneimine (PEI)-poly(ethylene glycol) (PEG)	Thermal	CTLA-4 and PD-1 blocking antibodies	N/A	4T1 tumor-bearing BALB/c mice	Breast cancer	[76]
	Dodecyl-modified hydroxypropyl methylcellulose (HPMC-C_12_) and biodegradable nanoparticles comprising poly(ethylene glycol)-*b*-poly(lactic acid) (PEG-PLA NPs)	N/A	CAR-T cells	N/A	MED8A tumor-bearing NSG mice	Medulloblastoma	[77]
	Hyaluronic acid	N/A	CAR-T cells, cytokine interleukin-15, and platelets conjugated with anti-PD-L1 blocking antibody	N/A	WM115 tumor-bearing NSG mice	Melanoma	[48]
	Paclitaxel fibers	N/A	aCD47 and Paclitaxel	Chemotherapy	GL-261 tumor-bearing C57BL/6 mice	Glioblastoma	[78]
	Cyclodextrin-polyethylene glycol	N/A	Cytosine-phosphate-guanine (CpG) nanoadjuvant	N/A	B16 tumor-bearing C57BL/6 mice	Melanoma	[50]
Immuno-therapy	Hyaluronic acid methacrylate (HAMA) with 2-(3-(6-methyl-4-oxo-1,4-dihydropyrimidin-2-yl)ureido)ethyl methacrylate (UPyMA) and diethylene glycol methacrylate (DEGMA)	N/A	DPPA-1 peptide (a d-peptide antagonist with a high binding affinity to programmed cell death-ligand 1 (PD-L1)) and doxorubicin	Chemotherapy	CT26 tumor-bearing BALB/c mice	Colorectal cancer	[79]
	Pluronic^®^ F127 and xanthan gum	Thermal	Granulocyte-macrophage colony stimulating factor	N/A	CT26 tumor-bearing BALB/cJRj mice	Colorectal cancer	[80]
	α-cyclodextrin/polyethylene glycol	N/A	Doxorubicin CpG self-crosslinking nanoparticles	Chemotherapy	B16 tumor-bearing C57BL/6 mice	Melanoma	[81]
	Pluronic^®^ F127 and gelatin	Thermal	Vemurafenib and anti-PD-1 antibody	Chemotherapy	D4M tumor-bearing C57BL/6 mice	Melanoma	[82]
	Four-arm polyethylene glycol thiol (PEGSH) and poly (ethylene glycol) diacrylate (PEGDA)	N/A	Doxorubicin and imiquimod (R837)	N/A	B16F10 tumor-bearing C57BL/6 mice	Melanoma	[83]
	Vitamin C amphiphile	N/A	Stimulator of interferon genes (STING) agonist-4 (SA)	N/A	CT26 tumor-bearing BALB/c mice	Colorectal cancer	[84]
	Hyaluronic acid and N-(3-Aminopropyl) methacrylamide hydrochloride-N-[Tris(hydroxymethyl) methyl]acrylamide- *p*(APMA-THMA)	N/A	Doxorubicin and kynureninase	Chemotherapy	B16F10 tumor-bearing C57BL/6 mice and 4T1 tumor-bearing BALB/c mice	Cancer	[85]
	Pluronic^®^ F127	Thermal	Sodium bicarbonate	N/A	MC38 tumor-bearing C57BL/6 mice	Colorectal cancer	[86]
	Dibenzaldehyde-functionalized polyethylene glycol (DF-PEG) and silk-chitosan	pH	Doxorubicin and JQ1	Chemotherapy	4T1 tumor-bearing BALB/c mice	Breast cancer	[87]
	Polyvinyl alcohol (PVA) crosslinked with N1-(4-boronobenzyl)-N3-(4-boronophenyl)-N1,N1,N3,N3- tetramethylpropane-1,3-diaminium (TSPBA)	Reactive oxygen species	IPI549 (PI3 kinase inhibitor) and anti-PD-L1 antibody	Chemotherapy	CT26 tumor-bearing BALB/c mice	Colorectal cancer	[88]
	Gellan gum	Light	Dawson-type (P_2_Mo_18_) polyoxometalate (POM) and Toll-like receptors agonist resiquimod (R848)	Phototherapy	4T1 tumor-bearing BALB/c mice	Breast cancer	[89]
	Pluronic^®^ F127	Thermal	TLR 7/8 dual agonist (MEDI9197)	N/A	B16-OVA tumor-bearing C57BL/6J mice	Melanoma	[90]
	Hydroxypropyl cellulose	Light	Interferon-α2b and cytokine-induced killer (CIK) cells	Radiotherapy	MKN-45 tumor-bearing BALB/c mice	Gastric cancer	[91]
	Tetra-armed PEG succinimidyl succinate (Tetra-PEG-SS) crosslinked with alkalescent bovine serum albumin	N/A	PQ912	N/A	B16F10 tumor-bearing C57BL/6 and 4T1 tumor-bearing BALB/c mice	Melanoma and Breast Cancer	[92]
	Supramolecular nanofibrils of thymopentin	Light	Thymopentin (TP5) and indocyanine green (ICG)	Phototherapy	Pan02 tumor-bearing C57BL/6 mice	Pancreatic cancer	[93]
	Sodium alginate	N/A	Toll-like receptor (TLR) agonists (CpG ODNs)	N/A	4T1 tumor-bearing C57BL/6 mice	Breast cancer	[94]
	CpG DNA	Enzymes, Light	Melanin and STING	Phototherapy	CT26 tumor-bearing Balb/c mice	Colorectal cancer	[95]
	Multimodule DNA	N/A	PD-1 aptamer, CpG ODN, captured T-cells	N/A	B16 tumor-bearing C57BL/6 mice	Melanoma	[96]
	Graphene oxide (GO) and polyethylenimine (PEI)	N/A	Messenger RNA and adjuvants (R848)-laden	N/A	B16 tumor-bearing C57BL/6 mice	Melanoma	[97]
	DNA CpG hydrogel, coated with melanin	Thermal and Light	Bis-(3^′^-5^′^)-cyclic dimeric guanosine monophosphate (G/DH)	Phototherapy	CT26 tumor-bearing C57BL/6 mice and 4T1 tumor-bearing C57BL/6 mice	Cancer	[95]
	PEG and Acrylated Sulfamethazine derivatives	pH and Thermal	Oncolytic adenoviruses	N/A	H1975 tumor-bearing mice	Lung cancer	[98]
Gene Therapy	Hyaluronic acid functionalized with levodopa and poly(ecaprolactone-co-lactide) ester	Thermal	GM-CSF & OVA-expressing Plasmid	Immunotherapy	B16 tumor-bearing C57BL/6 mice	Lung cancer and Melanoma	[99]
	Alginate-g-poly(N-isopropylacrylamide) (PNIPAAm)	Thermal	RALA/pDNA NPs	N/A	PC3 and MG63 cells	Castrate-resistant Prostate cancer	[100]
	Pluronic^®^ F127 and poly(2-dimethylamino)ethyl methacrylate-b-poly[(R)-3-hydroxybutyrate] (PDMAEMA-b-PHB)	Thermal	Survivin antisense oligonucleotide (Sur-ASON)	N/A	MCF-7/PDR tumor-bearing BALB/c mice	Breast cancer	[101]
	RNA-triple-helix consisting of one tumour suppressor miRNA (miRNA-205) and one oncomiR inhibitor (miRNA-221)	N/A	siRNA duplexes of CXCR4 LXL-DNA aptamer	N/A	MDA-MB-231 tumor-bearing BALB/c mice	Breast Cancer	[102]
	Gelatin	N/A	Recombinant Adeno-associated virus	N/A	C57BL/6 mice	Glioblastoma	[103]

Note: Abb: N/A, not applicable; NIR, near-infrared; PEI, poly(ethyleneimine); PEG, poly(ethylene glycol); PEG-PLA, poly(ethylene glycol)-block-poly(lactic acid) nanoparticles; CAR-T, chimeric antigen receptor T cells; PD-1, programmed cell death protein 1; CTLA-4, cytotoxic T-lymphocyte-associated protein 4; STING, stimulator of interferon genes; CpG ODNs, cytosine-phosphate-guanine oligodeoxynucleotides; CIK, cytokine-induced killer cells; GO, graphene oxide; RALA/pDNA NPs, RALA peptide/plasmid DNA nanoparticles; PNIPAAm, poly(N-isopropylacrylamide); PDMAEMA-b-PHB, poly(2-dimethylaminoethyl methacrylate)-block-poly[(R)-3-hydroxybutyrate]; Sur-ASON, survivin antisense oligonucleotide; siRNA, small interfering RNA; CXCR4, C-X-C chemokine receptor type 4; AAV, adeno-associated virus; G/DH, bis-(3^′^-5^′^)-cyclic dimeric guanosine monophosphate.

## 3 Hydrogels for Local Delivery of Monoclonal Antibodies

Therapeutic antibodies are a class of biological drugs that are designed to specifically bind to target molecules, such as proteins or receptors, involved in disease processes. These antibodies have revolutionized the field of medicine, particularly in the treatment of cancer and autoimmune diseases [36]. Incorporating therapeutic antibodies into hydrogels is an emerging strategy that offers unique advantages for drug delivery and localized therapy. Hydrogels can provide a protective environment for therapeutic antibodies, shielding them from degradation and enzymatic activity, and promoting their sustained release over time [37]. Also, due to the hydrogels’ soft and aqueous properties, they can maintain the antibody structure.

Some of the earliest studies describing the use of hydrogels for antibody-controlled release appeared in the 1990s by exploring poly(ethylene-co-vinyl acetate)-based hydrogels for brain and vaginal local delivery of IgG [38,39]. Over the years, other works explored the utility of hydrogels for the delivery of antibodies applied to many clinical situations, including wet age-related macular degeneration [40] or cancer.

**Figure 2:**
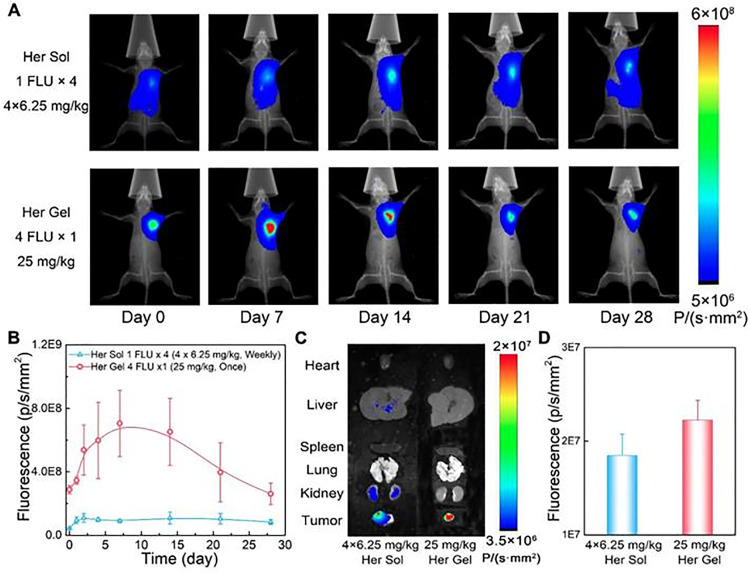
*In vivo* behavior of the Trastuzumab-loaded mixture-A hydrogel (Her Gel). (**A**) Real-time *in vivo* fluorescence imaging of SK-BR-3 tumor-bearing nude mice after hypodermic injection of Cy5.5-Her solution (Her Sol) or Cy5.5-Her-loaded mixture-A hydrogel. The solution was administered weekly for 4 weeks, whereas the hydrogel was injected once, resulting in an equivalent total Cy5.5-Her dose. Images within each group were obtained from the same mouse. A higher fluorescence intensity is seen in animals receiving the hydrogel compared to the solution (**B**) Semi-quantitative analysis of fluorescence intensity at the injection sites over time. (**C**) *Ex vivo* fluorescence imaging of major organs and tumors collected post-treatment (day 28). (**D**) Semi-quantitative analysis of tumor fluorescence on day 28. Reprinted from Ref. [37] under Creative Commons Attribution (CC BY) license, Copyright 2023 Ivyspring International Publisher

The work of Chen et al. is an example of the application of hydrogels for the sustained release of antibodies for cancer therapy [37]. In this work, physically crosslinked poly(lactic acid-co-glycolic acid)-b-poly(ethylene glycol)-b-poly(lactic acid-co-glycolic acid) (PLGA-PEG-PLGA)-based thermosensitive hydrogels were developed for the delivery of Trastuzumab, a HER2-targeting monoclonal antibody, to prevent the local relapse of HER2+ breast tumors, and to minimize the systemic-adverse effects such as the hematological and gastrointestinal toxicity. To achieve thermoresponsive properties, a blending of two copolymers with solubilities through different molecular weights of PLGA and PEG chains (1505–1000–1505 g/mol for copolymer 1 and 1250–1500–1250 g/mol for copolymer 2). The performance, degradation rate, and drug release kinetics of hydrogels were easily adjustable by simply varying the mix proportion of the copolymers. The formulation with a ratio of copolymer 1: copolymer 2 of 7:3 and a 25 wt% polymer in water presented the best release profile, allowing a sustained release of Trastuzumab *in vitro* for up to 80 days. Moreover, in a persistence/degradation study *in vivo*, after a subcutaneous injection, the hydrogel persisted for 4–5 weeks. On the other hand, a formulation with a more hydrophilic nature obtained by mixing copolymer 1 and 2 in an equal ratio presented a 90% release in 30 days and degraded in 3 weeks, demonstrating that the molecular weight and the nature of the polymer influence its behavior. Regarding *in vivo* performance, an intratumoral antibody accumulation was observed after a single hypodermic administration (Fig. 2). Consequently, the designed formulation demonstrated anti-relapse therapeutic efficacy and reduced the risk of cardiotoxicity, in comparison with Trastuzumab solutions. Many antibodies used in cancer therapy fall in the cancer immunotherapy category, and for this reason, are presented in the following section. Other studies applying hydrogels for local delivery of antibodies are depicted in Table 1.

## 4 Hydrogels for Local Delivery of Immunotherapeutic Agents

In the last decades, immunotherapy has received increasing attention and has been responsible for significant progress in the cancer treatment field, presenting very promising results both for the treatment of primary and metastatic tumors. For this reason, cancer immunotherapy was considered the scientific breakthrough of the year in 2013 by Science magazine [41]. Cancer immunotherapy aims to activate and enhance the body’s immune system to recognize and attack cancer cells. To achieve this aim, different strategies have been proposed, namely the use of immunomodulators (immune checkpoint inhibitors, antibodies, cytokines, etc.), cellular immunotherapy, and cancer vaccines [42]. The challenges related to the intravenous administration of immunotherapeutics, such as the limited immune responses and the severe off-target and secondary effects (which oblige the use of low doses), encourage the development of new approaches based on local treatment, with a special focus on hydrogels [43,44]. In this sense, many researchers have been working on the development of hydrogel-based platforms for local immunotherapy using different compositions, including synthetic polymers, polysaccharides, nucleic acids, peptides, proteins, and hybrids [44]. Examples of hydrogel-based local immunotherapies are depicted in the following sections.

### 4.1 Immune Checkpoint Inhibitor Delivery

Immune checkpoint inhibitors (ICIs) are monoclonal antibodies that block inhibitory signals in the immune system to enhance anti-tumor immunity [42]. Different clinical trials and clinical evidence have proven the potential of ICIs in cancer treatment [45]. Such results could be improved by the local administration of ICIs. Hydrogels can be used to deliver ICIs directly to the tumor microenvironment, increasing the local concentration and reducing systemic toxicity. To our knowledge, these systems are only at the preclinical phase; it is expected that some formulations will enter clinical trials in the next years.

**Figure 3:**
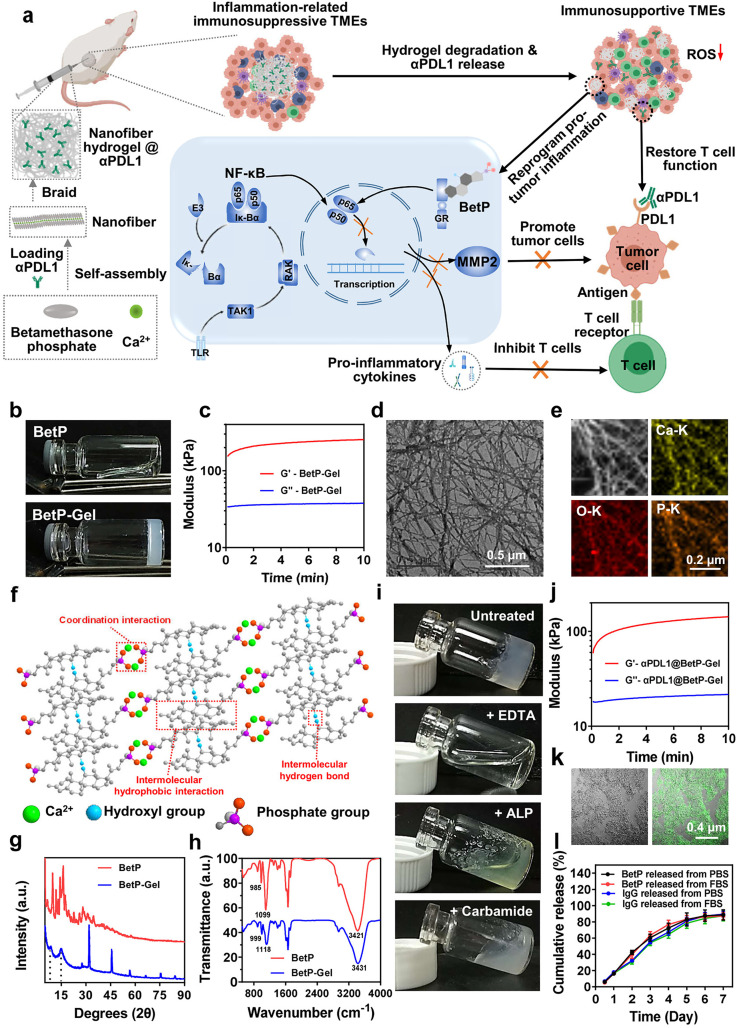
The formation and characterization of a nanofiber anti-inflammatory hydrogel. (**a**) Schematic of nanofiber hydrogel formation via cross-linking filamentous assemblies through physical interactions between betamethasone phosphate and Ca^2+^. This hydrogel can both reprogram the immunosuppressive tumor microenvironment by inhibiting NF-κB signaling and sustainably release αPDL1 to activate T cells, synergistically enhancing antitumor immunity. (**b**) Photographs of BetP solution (without Ca^2+^) and hydrogel (with Ca^2+^). (**c**) Rheological behavior of BetP hydrogel. G^′^, storage modulus; G^″^, loss modulus. (**d**) Representative TEM image of the hydrogel; (**e**) Element mapping of BetP hydrogel. (**f**) Ball-and-stick model showing intermolecular noncovalent interactions: coordination, hydrophobic, and hydrogen bonds. (**g**,**h**) XRD patterns and FTIR spectra of BetP powder and hydrogel. (**i**) Hydrogel stability after addition of EDTA (50 mM), ALP (1 μM), and carbamide (1 M). (**j**) Rheological behavior of BetP hydrogel encapsulated with αPDL1. G^′^, storage modulus; G^″^, loss modulus. (**k**) Representative fluorescence images of αPDL1-loaded hydrogel (αPDL1 stained with Alexa Fluor 488 conjugated anti-mouse antibody). (**l**) Cumulative release profiles of BetP and IgG from BetP hydrogel in PBS or PBS + 10% FBS over 7 days at 37°C (mean ± SEM, n = 3). Reprinted with permission from Ref. [46], Copyright 2023, American Chemical Society

For example, Chen et al. developed a carrier-free system based on a hydrogel composed of the self-assembled nanofibers of betamethasone phosphate, an anti-inflammatory drug that has the capacity to reprogram the pro-tumoral immunosuppressive tumor microenvironment to an antitumoral microenvironment, crosslinked with calcium chloride (Fig. 3) [46]. Moreover, the system was designed to serve as a reservoir for the sustained delivery of the anti-programmed cell death protein ligand 1 (αPDL1), an ICI to boost the immune system. The formulation presents all the physicochemical properties to be injected and forms a depot system for the controlled release of αPDL1 for one week (Fig. 3). With the local injection of the αPDL1-loaded hydrogel, effective therapeutic effects were observed in inhibiting both local tumors and abscopal tumors [46].

### 4.2 CART-Cell Therapy in Hydrogels

Chimeric antigen receptor (CAR) T-cell therapy involves genetically engineering a patient’s T-cells to recognize and attack cancer cells. Hydrogels can be used to encapsulate CAR T-cells and deliver them directly to the tumor site. This approach has shown promise in preclinical studies for the treatment of solid tumors [47].

The work of Hu et al. describes the development of a hydrogel-based approach for inhibiting post-surgery tumor recurrence [48]. The hydrogel was composed of acrylate-group-modified hyaluronic acid crosslinked by ultraviolet irradiation with N,N-methylenebisacrylamide using Irgacure 2959 as a photo-initiator. The approach involves the local delivery of CAR-T cells and platelets conjugated with an anti-programmed cell death ligand-1 (PDL1) antibody, using a hydrogel platform. The effectiveness of the hydrogel system was evaluated in a mouse model of postsurgical tumor recurrence. The results showed that the hydrogel system was effective in localizing the CAR-T cells and anti-PDL1-conjugated platelets at the tumor site, resulting in a significant reduction in tumor recurrence. The study also demonstrated that the hydrogel system was able to provide sustained release of the therapeutic agents, thereby prolonging their therapeutic effect. The study suggests that the hydrogel-based approach has potential as a strategy for inhibiting post-surgery tumor recurrence and highlights the importance of localized delivery of immunotherapeutic agents for improved efficacy in cancer treatment [48].

### 4.3 Cancer Vaccines in Hydrogels

Unlike preventive vaccines that aim to prevent infections, cancer vaccines are designed to treat existing cancer or prevent cancer recurrence. The fundamental principle behind cancer vaccines is to activate the immune system, particularly T cells, to recognize cancer cells as foreign or abnormal and mount an immune response against them. This immune response can involve various mechanisms, including the production of cytotoxic T lymphocytes (CTLs) that directly kill cancer cells, the activation of other immune cells to attack the tumor, and the production of antibodies that target cancer-specific antigens [49].

There are different types of cancer vaccines, including: (i) peptide or protein-based vaccines, (ii) whole-cell vaccines, (iii) DNA or RNA-based vaccines, and (iv) viral vector-based vaccines.

**Figure 4:**
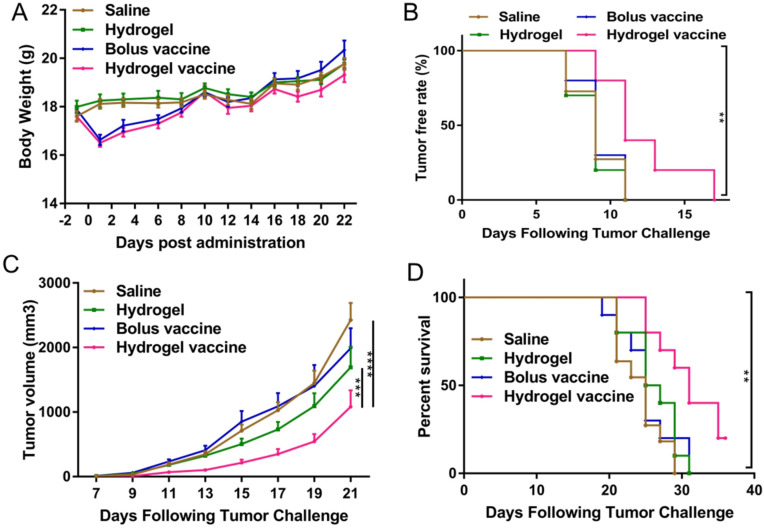
Prophylactic efficacy of the hydrogel vaccine in C26 tumors. (**A**) Change in mouse body weight post-administration. (**B**) Tumor-free rate after tumor challenge. (**C**) Tumor volume changes over time. (**D**) Percentage survival of Balb/c mice prophylactically vaccinated with different formulations. Data represent means ± SEM (n = 10–11). Data were analyzed using a two-way analysis of variance (ANOVA, **p < 0.01, ***p < 0.001, ****p < 0.0001). Reprinted with permission from Ref. [51], Copyright 2023, Elsevier

Cancer vaccines are an active area of research and clinical development. While some cancer vaccines have shown promising results in clinical trials, their widespread use as a standalone therapy has been limited. Currently, cancer vaccines are often used in combination with other immunotherapies, such as immune checkpoint inhibitors or immune system modulators, to enhance their efficacy and produce better treatment outcomes [49,50], being formulated in different systems, including hydrogels. As an example, Yang et al. developed an injectable and biodegradable thermosensitive hydrogel vaccine composed of physically crosslinked poly(d,l-lactide)-poly(ethylene glycol)-poly(d,l-lactide) (PDLLA-PEG-PDLLA) encapsulating granulocyte-macrophage colony-stimulating factor (GM-CSF), TLR 9 agonist CpG-ODN, and tumor cell lysates (TLs) [51]. The obtained results demonstrate that the sustained release of immunomodulators and antigens promoted by the hydrogel-based vaccine significantly activates and matures dendritic cells both *in vitro* and *in vivo*. Moreover, a tumor suppression and prolonged overall survival time were observed in both prophylactic and therapeutic experiments (Fig. 4) [51].

**Figure 5:**
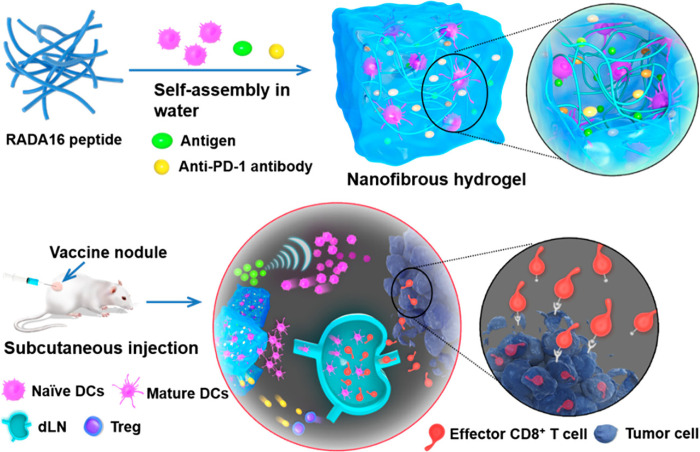
Schematic structure and mechanism of action of the dendritic cells (DCs)-based vaccine formulated in a peptide nanofibrous hydrogel. RADA16 peptides self-assemble into a nanofibrous hydrogel containing exogenous DCs, antigens, and anti-PD-1 antibodies. After injection, the vaccine releases exogenous DCs and antigens that recruit the host DCs, activating endogenous DCs. Both mature DCs migrate to the draining lymph nodes (dLN) and amplify the antigen-specific T-cell immunity. Finally, CD8^+^ effector T cells infiltrate the tumor tissue and kill tumor cells. Moreover, the anti-PD-1 antibodies block the PD-1 pathway, decrease the production of regulatory T cells (Tregs), and reduce the tumoral immunosuppression environment. Reprinted with permission from Ref. [52], Copyright 2023, American Chemical Society

In another study, Yang et al. developed a hydrogel composed of the self-assembling of RADA16 peptide (Ac-RADARADARADARADARADA-CONH2) nanofibers loading dendritic cells, an anti-PD1 antibody, and ovalbumin as a model antigen [52]. The hydrogel maintained the viability and biological function (antigen uptake and maturation) of encapsulated dendritic cells and simultaneously recruited a number of host dendritic cells. This promoted the drainage of activated dendritic cells to lymph nodes, resulting in a potent cellular immune response (Fig. 5).

**Figure 6:**
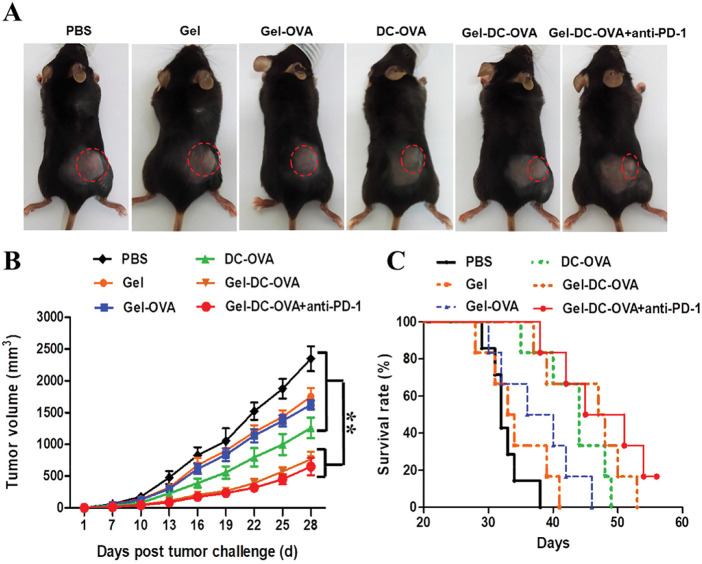

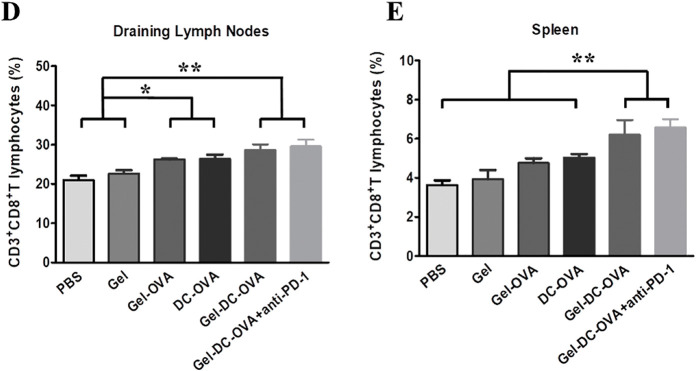
Vaccine nodules exhibited antigen-specific protective antitumor response. (**A**) Representative tumors were observed on day 14 after the inoculation of tumor cells. (**B**) The tumor volume within the 28-day period after tumor cell inoculation. Day 0 represents the first day of the tumor challenge. (**C**) The survival rate of mice. (**D**,**E**) The percentage of CD3^+^ and CD8^+^ T cells in the draining lymph nodes (**D**) or in the spleen (**E**) of vaccinated mice was determined on day 28 after the tumor challenge. Data are shown as mean ± SDs (n = 6) from at least three independent experiments. *p < 0.05, **p < 0.01. Reprinted with permission from Ref. [52], Copyright 2023, American Chemical Society

The developed hydrogel-based vaccine, designed for combined therapy using dendritic cells, immunomodulators, and immune checkpoint inhibitors, presented a superior activation of the immune response, a decrease in the tumor volume, and a significant increase in the survival of the animals treated, compared to the hydrogels containing monotherapy (Fig. 6) [52].

### 4.4 Combined Therapy in Hydrogels

Immunotherapy can be used as monotherapy or, more commonly, combined with chemotherapy, radiotherapy, and phototherapy.

#### 4.4.1 Chemo-Immunotherapy

Chemo-immunotherapy is a treatment approach that involves the simultaneous or sequential administration of chemotherapy drugs with immunotherapeutic agents. It takes advantage of the unique mechanisms of action and the synergistic effects of both therapies, showing particular success in certain types of cancer, such as lung cancer, melanoma, and lymphoma. Chemo-immunotherapy represents an evolving and promising field in cancer treatment, offering new avenues to enhance the effectiveness of traditional chemotherapy by harnessing the power of the immune system [53]. Ongoing research and clinical trials continue to explore and refine the optimal combinations and strategies for chemo-immunotherapy in various cancer types.

For example, Wang et al. built a prodrug hydrogelator for the local delivery of camptothecin and an immune checkpoint blocker, the aPD1 antibody, to improve the host’s immune system against tumor [54]. The hydrogelator was composed of a peptide diCPT-PLGLAG-iRGD (CRGDRGPDC conjugated with RGD) that self-assembles into nanotubes, forming a hydrogel. The obtained *in vivo* results demonstrate a strong and durable systemic anticancer immunity, with a consequent tumor regression and inhibition of tumor recurrence and metastasis [54]. These results claim the importance of chemo-immunotherapy in the design of innovative anti-cancer strategies.

#### 4.4.2 Radioimmunotherapy

In this chapter, the term radioimmunotherapy refers to the combined administration of radiotherapy and immunotherapy [55,56]. Hydrogels can be used to deliver radioimmunotherapy directly to the tumor site, enabling targeted radiation and sustained delivery of immunotherapy. This approach can be especially useful for sensitive tumors to radiotherapy and overcome the development of radio-resistance.

For example, Zhang et al. developed a hydrogel based on the self-assembly of nanofibers of the toll-like receptor agonist TLR7/8a conjugated with a radiosensitive peptide (Smac N7) through succinic acid as a linker [57]. The Smac-TLR7/8 hydrogel was easily locally injected in the tumor site and, after γ-ray radiation, polarized the macrophages M2 into M1 type. This repolarization, in combination with radiotherapy, led to an increase in the tumor necrosis factor secretion, which activated the antitumor immune response and effectively inhibited tumor growth. Moreover, the repolarization of macrophages alters the tumor microenvironment, enhancing the efficacy of immune checkpoint blockage [57].

The promising results of hydrogels for local radioimmunotherapy gave rise to a formulation that recently enrolled in clinical evaluation regarding its safety (Phase 1) to treat unresectable colorectal liver metastases. The undisclosed thermoreversible hydrogel formulation was developed to deliver GM-CSF and mifamurtide [58].

#### 4.4.3 Photothermal-Immunotherapy

Photothermal-immunotherapy is a combination therapy that integrates photothermal therapy (PTT) and immunotherapy to enhance the treatment of cancer. It is a promising approach that combines the localized heat generation of photothermal agents with the immune-stimulating effects of immunotherapy to achieve synergistic therapeutic outcomes [59]. Photothermal agents, typically nanoparticles often made of materials such as gold, carbon nanotubes, or graphene, can absorb specific wavelengths of light and convert the light energy into heat through a process called plasmonic photothermal conversion. When exposed to laser light at the appropriate wavelength, these nanoparticles generate heat, leading to localized hyperthermia in the target tissue [60]. In photothermal-immunotherapy, the heat generated by photothermal agents during PTT serves multiple purposes. Firstly, localized hyperthermia can directly induce tumor cell death by damaging cellular structures and triggering apoptosis (programmed cell death). Secondly, the heat can disrupt the tumor microenvironment, increasing the accessibility of immune cells to the tumor site and facilitating immune responses [59].

In a study by Jia et al., a physically crosslinked PDLLA-PEG-PDLLA hydrogel incorporating nanoparticles containing indocyanine green (photothermal agent), resiquimod (TLR-7/8 agonist), and CPG ODNs (TLR-9 agonist) [61]. The hydrogel is locally injected into the tumor resection cavity and, after near-infrared stimulation, releases the therapeutic compounds that, in combination with the photothermal effect, ablate residual tumor tissues and produce tumor-associated antigens. This system can achieve synergistic photothermal immunotherapy and prevent breast cancer postoperative relapse [61].

## 5 Hydrogels for Local Delivery of Gene Therapy

Gene therapy involves introducing genetic material into cells to treat or prevent diseases. The major challenge regarding gene therapy is related to the fast degradation of the oligonucleotides and their inability to enter the cells without an adequate vector. Although viral vectors are still the most commonly used, their immunogenic adverse effects urged the need to find efficient non-viral vectors for gene therapy [62]. Hydrogels are a promising platform to deliver gene therapies directly to the tumor site in order to achieve the desired gene overexpression and/or gene silencing. Another therapeutic approach would consist of the use of hydrogels to deliver gene therapies that sensitize cancer cells to chemotherapy drugs or enhance the immune response [63]. Some examples of gene therapy effectors that are being delivered by hydrogels include:

### 5.1 DNA

**Figure 7:**
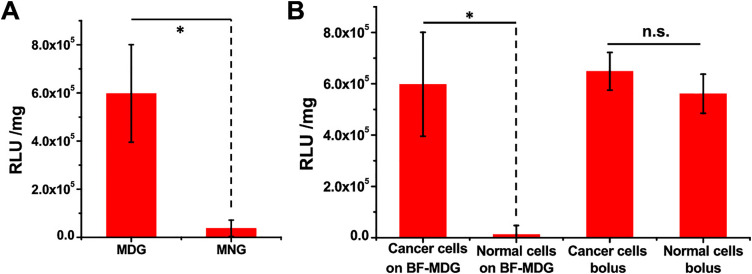
Surface-mediated stimuli-responsive and differentiated gene transfection on breath figure matrix metalloproteinase (MMP)-degradable gel cross-linked by the MMP-sensitive peptide (BF-MDG) evaluated by luciferase activity (RLUs per mg protein, RLU/mg protein). (**A**) Transgene expression levels of MDA-MB-231 cells on BF-MDG or breath figure MMP-nondegradable gel cross-linked by the MMP-insensitive peptide (BF-MNG). (**B**) Transgene expression levels of MDA-MB-231 (cancer cells) and HBL-100 (normal cells) by BF-MDG surface-mediated transfection and traditional bolus transfection. *p < 0.05, ns no significance. Reprinted with permission from Ref. [65], Copyright 2023, American Chemical Society

DNA in the form of plasmids can be used to deliver therapeutic genes to cancer cells, such as tumor suppressor genes or genes that induce cell death [64]. Controlled and localized delivery is required to promote higher transfection efficiencies and specificity. Hydrogels can be great tools to deliver DNA directly to the tumor site, enabling sustained and prolonged gene expression. One example of this is the work of Zhang et al., who developed an enzymatic-responsive platform for the release of DNA [65]. This platform consists of an MMP-responsive hydrogel that was then loaded into the surface of a breath figure (BF) porous film, forming an *in situ* DNA reservoir. The hydrogel was composed of four-armed poly(ethylene glycol) (PEG) acrylate crosslinked with a MMP-sensitive peptide (GCRD-GPQGIWGQ-DRCG), loaded with DNA:branched poly(ethyleneimine) (PEI) polyplexes. Since the presence of MMP in cancer is significantly higher than in normal cells, the developed formulation releases the DNA in response to cancer cells and specifically transfects them [65] (Fig. 7). This strategy opens a new window of possibilities in the field of localized gene therapy.

### 5.2 RNA Interference

**Figure 8:**
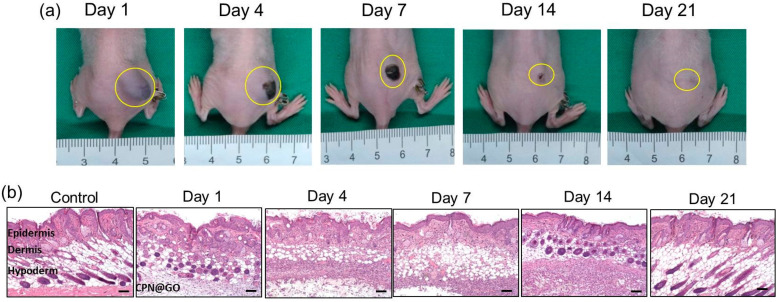
The *in vivo* degradation of graphene oxide-loaded chitosan-g-poly(N-isopropylacrylamide) (CPN@GO) hydrogel after the subcutaneous injection of a polymer solution containing 10% (w/v) CPN and 0.5% (w/w) GO to the right flank of a nude mouse. Gross view images were taken at different time points post-implantation (**a**), and the tissues surrounding the hydrogels were dissected and subject to hematoxylin–eosin stain (**b**), scale not indicated in the original publication. Reprinted from Ref. [67] under Creative Commons Attribution (CC BY) license, Copyright 2023 MDPI

RNA interference (RNAi) is a biological process in which RNA molecules inhibit the expression of specific genes. It is a powerful mechanism that regulates gene expression and plays a crucial role in various biological processes. The RNAi pathway involves the use of small RNA molecules, specifically small interfering RNA (siRNA), microRNA (miRNA), or short-hairpin RNA (shRNA) to target and degrade messenger RNA (mRNA) molecules that encode for specific proteins of interest. This interference can result in the transitory suppression or silencing of the targeted gene, leading to a reduction of the corresponding protein’s expression [66]. Given the high instability of these types of molecules, they need a vector that protects their integrity and delivers them into the cells of interest. The use of hydrogels for the delivery of this type of molecule can bring the opportunity to promote a prolonged reduction in the expression of a certain oncogene, consequently impairing tumor development. For example, in the study of Lu et al., a multifunctional chemo/gene delivery system was developed for the treatment of glioblastoma multiforme [67]. The proposed formulation combined ligand-mediated active targeting and pH-triggered drug release features. Briefly, the thermosensitive-based hydrogels composed of chitosan-g-poly(N-isopropylacrylamide) polymer (CPN) served as a platform for the sustained release of stomatin-like protein 2 (SLP2) short hairpin RNA (shRNA) and a complex formed by the irinotecan (CPT-11) and the cetuximab (CET)-conjugated graphene oxide (GO) (GO-CET/CPT11). The hydrogel demonstrated an adequate, sustained, and controlled release of the compounds and slowly degraded over 3 weeks (Fig. 8). Moreover, it significantly inhibits tumor malignancy both *in vitro* and *in vivo* in glioblastoma models [67].

### 5.3 CRISPR-Cas9 Gene Editing

CRISPR-Cas9 is a gene editing technology that can be used to modify the DNA of cancer cells, promoting the knockdown of the genes of interest [68]. Hydrogels can be used to deliver CRISPR-Cas9 components directly to the tumor site, enabling precise and targeted gene editing and reducing the risk of off-target effects.

For example, the work of Chen et al. consists of the development of liposome-templated hydrogel nanoparticles, formed by the physical crosslinking and self-assembly of liposomes, for the delivery of Cas9 protein and nucleic acids [69]. The developed formulation demonstrated higher transfection efficiency than the gold standard Lipofectamine^®^ 2000, serving as an efficient platform for the targeted inhibition of genes in tumor cells. Since this work is focused on the treatment of brain tumors, the proposed formulation was used for the delivery of CRISPR/Cas9 targeting the polo-like kinase 1 (PLK1). Consequently, significant inhibition of tumor growth and an improvement in mouse survival were observed in the *in vivo* experiments [69].

### 5.4 Oncolytic Virus

Oncolytic viruses are viruses that selectively replicate and kill cancer cells. The intratumoral injection of effector cells combined with an oncolytic adenovirus expressing antitumor cytokines is an effective strategy to promote an antitumor immune effect by oncolysis and by altering the tumor microenvironment [70]. In order to avoid the risk of immunogenicity, the possibility of oncolytic viruses spreading into other organs, and to avoid the repetitive administrations necessary to reach therapeutic doses, some strategies based on hydrogels are being developed. One example is the work of Du et al., who designed a gelatin-based hydrogel, composed of gelatin crosslinked with hydroxyphenylpropionic acid via 1-ethyl-3-(3-dimethylaminopropyl)-carbodiimide for the sustained delivery of oncolytic adenovirus armed with IL12 and IL15 (CRAd-IL12-IL15) and CIK cells, to promote prolonged antitumor effects after a single intratumoral injection [71]. This strategy significantly reduced the dispersion of the cargo to other organs, avoiding the occurrence of adverse effects. Moreover, the designed hydrogel treatment promoted a sustained release of the cargo over a longer period of time, being able to induce potent anti-tumor immune responses with a single administration [71].

## 6 Discussion

**Figure 9:**
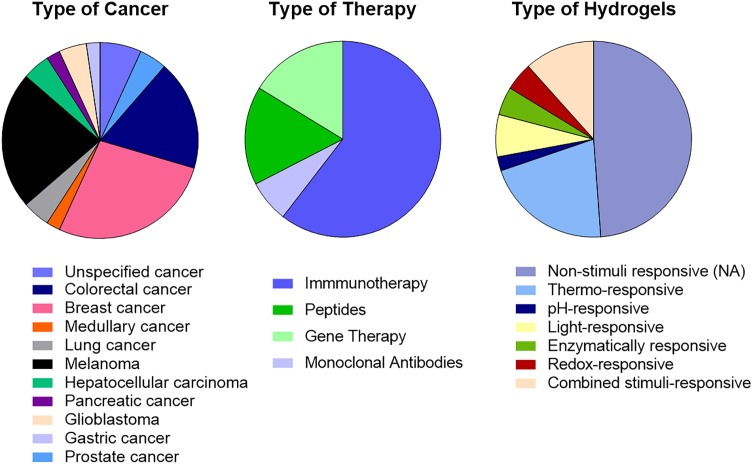
Comparative analysis of 43 hydrogel systems from the literature. The charts illustrate the predominant design choices and application areas guiding current research in hydrogel-based cancer treatment

This article sheds light on the remarkable versatility of hydrogels in enhancing the sustained and localized release of peptides, antibodies, immunotherapeutic agents, and gene therapies to treat cancer, with examples shown in Table 1 and summarized in Fig. 9. As observed, the majority of the hydrogels under development are designed for immunotherapy. Breast cancer and melanoma emerge as the most commonly studied tumor models, with additional interest in glioblastoma and colorectal cancer. Moreover, Thermo-responsive hydrogels constitute the largest material category, reflecting the widespread use of temperature-induced sol–gel transitions for minimally invasive administration

Hydrogel platforms are poised to transition from experimental concepts to clinical realities in oncology over the next decade. The ability of hydrogels to achieve both sustained and localized release of therapeutics, including peptides, antibodies, immunotherapeutic agents, and genetic material, marks an important advancement in precision medicine. By leveraging the exclusive properties, biocompatibility, and responsiveness to stimuli such as pH, temperature, or enzymatic activity, hydrogels can overcome key limitations of systemic administration, such as poor site-specific accumulation, instability, and dose-limiting toxicity.

Examination of the current literature reveals that there is no optimal hydrogel platform for the delivery of biopharmaceuticals and advanced therapies. The adequate formulation depends strongly on the physicochemical nature of the therapeutic cargo and the intended use. Hydrogels based on natural polymers such as chitosan, alginate, hyaluronic acid, and gelatin exhibit superior biocompatibility and intrinsic bioactivity, making them particularly suitable for the localized release of sensitive biomolecules such as antibodies, peptides, genetic material, or cells, where absent or mild crosslinking and aqueous processing prevent denaturation [104]. In contrast, synthetic hydrogels provide higher mechanical strength, tunable degradation rates, and predictable crosslinking density, advantages critical for drug delivery, which require controlled diffusion and are more stable [105]. Hybrid systems that integrate both natural and synthetic components have emerged as promising “best-of-both-worlds” designs, combining the mechanical stability of synthetic polymers with the biofunctionality of polysaccharides and other natural compounds [106,107]. Overall, natural hydrogels are more common for protein and cell delivery, while synthetic and hybrid platforms excel in gene delivery and chemotherapeutic release [108,109]. Synthesizing insights from published works, some design guidelines can be proposed: (1) select hydrogels with high water content and minimal crosslinking for cells and fragile biomolecules; (2) favor ionic hydrogels for nucleic acids; (3) tune degradation kinetics throw covalent crosslinking to match the intended therapeutic window; and (4) exploit stimuli-responsive mechanisms, such as pH or enzymatic sensitivity, to achieve on-demand release in specific disease conditions. These considerations underpin the growing shift toward modular and multifunctional hydrogel architectures in oncologic drug and biopharmaceutical delivery. Studies also demonstrate that the therapeutic performance of hydrogel systems is not only dependent on their composition but also on their microstructural architecture and crosslinking strategy. For instance, physically crosslinked hydrogels allow reversible, non-covalent interactions, providing better injectability and self-healing capacity, critical for intratumoral delivery. Conversely, covalently crosslinked systems afford superior control of release kinetics but may compromise the stability of fragile biologics if the reaction conditions are harsh [107,110].

One of the most promising possibilities is the integration of hydrogels into personalized medicine. The ability to customize hydrogel formulations to adapt to the unique genetic, molecular, and physiological profiles of individual patients could revolutionize cancer treatment. Personalized hydrogels could be designed to release specific biopharmaceuticals, immunotherapeutic agents, or genetic material in response to the unique tumor microenvironment, thereby maximizing therapeutic efficacy and minimizing adverse effects. Among the different options, smart hydrogels that can respond to specific stimuli such as pH, temperature, or enzymatic activity represent a cutting-edge approach to controlled drug release. These hydrogels can be engineered to release their therapeutic payloads in response to the specific conditions of the tumor microenvironment, thereby ensuring that the drugs are delivered precisely where and when they are needed. In fact, by unveiling the mechanisms, strategies, and applications of hydrogel-mediated delivery, we unlock a future where medical interventions are not only more effective but also finely tailored to individual patient needs. Furthermore, hydrogels represent an excellent platform for combination therapies, as demonstrated in this chapter, wherein multiple therapeutic agents can be co-delivered to target different aspects of cancer pathology simultaneously. For example, hydrogels could be designed to release either chemo- and immunotherapeutic drugs or a combination of gene therapy vectors and anti-cancer drugs. This multi-therapy approach could enhance the overall therapeutic outcome and overcome resistance mechanisms that often undermine single-agent therapies. In this sense, a particularly effective design strategy involves the creation of multicomponent hydrogels through the incorporation of nanoparticles and other nanostructures into hydrogels, creating a wide range of possibilities in terms of cargo capacity and release rates. For example, micelles-in-hydrogel systems have shown a remarkable ability to modulate both hydrophilic and hydrophobic drug release rates, enabling spatiotemporal control of combination therapies [111]. In this context, integrating nanoparticles within hydrogel matrices can enhance drug loading and protection while reducing burst effects and diffusion losses [112].

## 7 Conclusions

Hydrogels have demonstrated exceptional versatility as platforms for sustained, localized, and stimuli-responsive delivery of peptides, antibodies, immunotherapeutic agents, and gene therapies for cancer treatment. Their unique ability to provide controlled release while minimizing systemic toxicity represents a major advancement in precision oncology.

Natural, synthetic, and hybrid hydrogels each offer distinct advantages. Natural polymers provide high biocompatibility and mild processing conditions suitable for fragile biomolecules, while synthetic hydrogels supply structural precision, reproducibility, and tunable degradation. Hybrid systems combine complementary strengths, creating increasingly sophisticated multifunctional architectures capable of addressing the diverse challenges of oncologic drug and biopharmaceutical delivery. Although no universally optimal hydrogel exists, rational design principles, considering therapeutic cargo properties, release requirements, and microenvironmental stimuli, enable the creation of tailored systems for specific cancer applications.

Despite significant progress, several critical challenges still limit the clinical translation of hydrogel-based platforms for cancer therapy. One major obstacle involves maintaining the stability and bioactivity of delicate macromolecules, including antibodies, enzymes, and RNA-based therapeutics, throughout formulation, storage, and release. Many of these molecules are sensitive to variations in pH, ionic strength, and temperature during hydrogel preparation or degradation. Crosslinking reactions, especially those involving reactive aldehydes or free radicals, can induce structural denaturation or loss of function, necessitating the use of mild, enzyme-mediated, or physical gelation approaches [108,113]. Additionally, the encapsulated drugs may adsorb onto the polymeric network or interact with charged residues, altering their release kinetics and reducing therapeutic efficacy. Another major challenge is the potential immunogenicity of natural hydrogels, particularly those derived from animal or microbial sources, such as chitosan or gelatin [114]. Batch-to-batch variability and residual impurities can elicit inflammatory or allergic responses, while synthetic hydrogels are more homogeneous and potentially less immunogenic [115]. Another critical point is the formulation’s biocompatibility. Hydrogel-cell interactions at the injection site critically determine both short- and long-term biocompatibility: material properties such as mesh size, stiffness, viscoelasticity, surface chemistry, and degradation products modulate cell adhesion, migration, proliferation, and phenotype (including macrophage polarization), and thereby influence drug release, cytocompatibility, tissue integration, and fibrotic encapsulation [116]. As commented, natural-based and physically crosslinked hydrogels preserve better the integrity of labile molecules and are expected to be more biocompatible; however, they may induce immune recognition and activation and permit faster immune cell infiltration. On the other hand, chemically crosslinked hydrogels may have tight cell diffusion but reduced cytocompatibility based on composition and reaction conditions [117]. *In vivo* immune responses to implanted hydrogels range from benign integration to acute inflammation and foreign-body reaction, outcomes that depend on polymer chemistry, impurities (batch variability), and the presence of bioactive motifs [116,118]. Establishing standardized purification and characterization protocols for natural polymers, endotoxin, and microbiological control is therefore essential to ensure reproducibility and regulatory compliance. The application of stimuli-responsive or smart hydrogels also faces practical hurdles. Although hydrogels responsive to pH, temperature, enzymes, or light are widely explored, their performance within the complex *in vivo* tumor microenvironment often deviates from *in vitro* predictions. Variations in pH gradients, enzymatic activity, and limited tissue penetration of external stimuli such as light can lead to incomplete or uncontrolled drug release [119]. Moreover, many smart hydrogels require external triggers or co-factors not easily applied in deep or irregular tumor tissues, limiting their clinical applicability. Overcoming these barriers requires a shift toward integrated design frameworks that couple computational modeling with experimental validation, allowing predictive control of hydrogel degradation, drug diffusion, polymer-cargo and polymer-cell interactions, and biological interactions. Future work should also focus on developing adaptive hydrogel systems that dynamically adjust their physicochemical behavior in response to the evolving tumor microenvironment [119].

## 8 Future Directions

To accelerate the clinical translation of this type of formulation, future research should prioritize personalized, multifunctional, and clinically translatable solutions.

Personalization will require tailoring hydrogel composition, mechanical properties, and release kinetics to the molecular, genetic, and physiological profiles of individual tumors. Adaptive hydrogels capable of responding dynamically to the evolving tumor microenvironment represent an important frontier, offering real-time modulation of drug release or degradation in response to endogenous cues. Equally important is multifunctionality, integrating therapeutic delivery with diagnostics, imaging, and on-demand release triggers. Such hydrogels could enable real-time monitoring of therapeutic distribution, release behavior, and treatment response, paving the way for next-generation theranostic systems. Another key direction is the development of combination therapies that co-deliver chemotherapeutics, immunomodulators, and genetic material to achieve synergistic effects. In parallel, attention must be given to regulatory pathways, with efforts focused on streamlining approval processes for hydrogel-based combination products while ensuring scalable and reproducible manufacturing. The clinical translation of hydrogel-mediated combination therapies faces notable regulatory hurdles due to the complexity of co-delivering multiple therapeutic agents [113]. Each component must meet safety, efficacy, and quality standards, while the combined system must demonstrate added therapeutic benefit without increased toxicity. Current regulatory frameworks, primarily designed for single-agent products, complicate the approval process for multimodal formulations. Developers must provide integrated preclinical and clinical data proving that the hydrogel platform maintains stable release kinetics, reproducibility, and biocompatibility, and that potential interactions among agents do not alter pharmacokinetics or pharmacodynamics. Early dialogue with agencies such as the FDA and EMA through regulatory guidance programs is recommended to align development strategies and facilitate approval. Finally, patient-centric design should remain at the forefront, emphasizing the creation of minimally invasive, injectable systems that maximize both comfort and ease of administration.

To these advanced hydrogel systems make a significant impact, overcoming regulatory and clinical translation challenges will be crucial. This includes rigorous testing to ensure biocompatibility, safety, and efficacy, as well as developing scalable manufacturing processes. Regarding this, quality control in scaling up production is a significant hurdle, as preclinical quality control methods may not be suitable for large-scale synthesis. This can lead to batch differences, reduced stability, poor therapeutic efficacy, and safety issues. For the specific case of smart hydrogels, such as thermosensitive, pH-sensitive, and photosensitive hydrogels, appropriate preparation and storage technologies are required for successful clinical translation. Importantly, hydrogels are classified as devices under the Federal Food, Drug, and Cosmetic (FD&C) Act and are considered combination products when drug-loaded, necessitating a lengthy FDA approval process that can exceed seven years, impacting commercial viability and increasing costs. Regarding the nature of the hydrogel components, so far, despite the advantages of natural polymers, only synthetic polymer-based hydrogels have received FDA approval, likely due to concerns about batch-to-batch variability and the limited supply of pharmaceutical-grade biopolymers. Lastly, the development of patient-centric hydrogel systems that prioritize ease of administration and patient comfort will be vital. Innovations such as injectable hydrogels that can be administered with minimal invasiveness, or hydrogels that can be monitored and adjusted post-application, could greatly enhance patient adherence and overall treatment experience. Collaborative efforts between materials scientists, oncologists, immunologists, and regulatory bodies will be essential to bring these advanced hydrogel systems from bench to bedside.

While hydrogel-mediated biomolecule delivery has made remarkable strides, there are still unexplored territories and challenges that lie ahead. Despite different hydrogels for localized anticancer therapy being under clinical evaluation, and some are already approved, they have mainly focused on chemotherapy. However, they demonstrate the therapeutic potential of hydrogel-based formulations, paving the way to their application in more advanced treatments such as immunotherapy and gene therapy. Up to now, only a few formulations to deliver biomolecules have reached clinical evaluation. Is the example of Alhydrogel^®^ vaccine with recombinant soluble prostate-specific membrane antigen (Rs-PSMA) or BIL06v peptide that completed its Phase I evaluation in prostate cancer treatment (NCT00705835) and advanced solid cancer (ACTRN12618000838213), respectively, or a Pluronic^®^ F127-based formulation for delivery of granulocyte-macrophage colony-stimulating factor and mifamurtide under Phase I evaluation for unresectable colorectal liver metastases (NCT04062721). Based on the results obtained so far with the developed formulations and the growing application of advanced therapies, the field of hydrogel-based delivery systems for localized therapy is expected to reach significant advancements and drive future innovations in several key areas, such as immunotherapy and gene therapy. Besides, many crucial aspects remain to be achieved, the evolution of this field continues to hold immense promise. Looking ahead, the field is expected to evolve rapidly through hybrid hydrogel platforms combining natural polymers for bioactivity with synthetic systems for mechanical precision. Such adaptive and modular hydrogels are poised to become central tools in personalized oncology, offering sustained, site-specific release of drugs, cells, antibodies, RNA therapies, and immunomodulators while minimizing systemic exposure. Predictive design approaches leveraging computational modeling, AI-assisted material optimization, and mechanistic understanding of tumor microenvironments will be essential to drive the next generation of hydrogel-based cancer therapies.

By uniting advances in biomaterials engineering with the expanding field of advanced cancer therapeutics, smart hydrogel systems have the potential to redefine, in the near future, localized cancer treatment, offering clinicians unprecedented control over therapeutic delivery and patients’ tangible improvements in survival and quality of life.

## Data Availability

The data supporting the information contained in this review are available within the cited references. No new datasets were generated or analyzed during the current study.

## References

[1] Dayanandan AP, Cho WJ, Kang H, Bello AB, Kim BJ, Arai Y, et al. Emerging nano-scale delivery systems for the treatment of osteoporosis. Biomater Res. 2023;27(1):68. doi:10.1186/s40824-023-00413-7; 37443121 PMC10347748

[2] Shan X, Gong X, Li J, Wen J, Li Y, Zhang Z. Current approaches of nanomedicines in the market and various stage of clinical translation. Acta Pharm Sin B. 2022;12(7):3028–48. doi:10.1016/j.apsb.2022.02.025; 35865096 PMC9293719

[3] Cheng Y, Zhang H, Wei H, Yu CY. Injectable hydrogels as emerging drug-delivery platforms for tumor therapy. Biomater Sci. 2024;12(5):1151–70. doi:10.1039/d3bm01840g; 38319379

[4] Andrade F, Durán-Lara E, Rafael D. Multicomponent hydrogels for cancer diagnosis and therapy. In: Dodda JM, Deshmukh K, Bezuidenhout D, editors. Multicomponent hydrogels: smart materials for biomedical applications. Cambridge, UK: Royal Society of Chemistry; 2023. p. 542–77. doi:10.1039/bk9781837670055-00542.

[5] Tenchov R, Bird R, Curtze AE, Zhou Q. Lipid nanoparticles—from liposomes to mRNA vaccine delivery, a landscape of research diversity and advancement. ACS Nano. 2021;15(11):16982–7015. doi:10.1021/acsnano.1c04996; 34181394

[6] Sosnik A, Seremeta KP. Polymeric hydrogels as technology platform for drug delivery applications. Gels. 2017;3(3):25. doi:10.3390/gels3030025; 30920522 PMC6318675

[7] Rafael D, Melendres MMR, Andrade F, Montero S, Martinez-Trucharte F, Vilar-Hernandez M, et al. Thermo-responsive hydrogels for cancer local therapy: challenges and state-of-art. Int J Pharm. 2021;606:120954. doi:10.1016/j.ijpharm.2021.120954; 34332061

[8] Thang NH, Chien TB, Cuong DX. Polymer-based hydrogels applied in drug delivery: an overview. Gels. 2023;9(7):523. doi:10.3390/gels9070523; 37504402 PMC10379988

[9] Zeimaran E, Pourshahrestani S, Fathi A, Bin Abd Razak NA, Kadri NA, Sheikhi A, et al. Advances in bioactive glass-containing injectable hydrogel biomaterials for tissue regeneration. Acta Biomater. 2021;136(1):1–36. doi:10.1016/j.actbio.2021.09.034; 34562661

[10] Gulrez SKH, Al-Assaf S, Glyn O. Hydrogels: methods of preparation, characterisation and applications. In: Progress in molecular and environmental bioengineering—from analysis and modeling to technology applications. London, UK: IntechOpen Limited; 2011. doi:10.5772/24553.

[11] Akhtar MF, Hanif M, Ranjha NM. Methods of synthesis of hydrogels ... A review. Saudi Pharm J. 2016;24(5):554–9. doi:10.1016/j.jsps.2015.03.022; 27752227 PMC5059832

[12] Ali F, Khan I, Chen J, Akhtar K, Bakhsh EM, Khan SB. Emerging fabrication strategies of hydrogels and its applications. Gels. 2022;8(4):205. doi:10.3390/gels8040205; 35448106 PMC9024659

[13] Lavrentev FV, Shilovskikh VV, Alabusheva VS, Yurova VY, Nikitina AA, Ulasevich SA, et al. Diffusion-limited processes in hydrogels with chosen applications from drug delivery to electronic components. Molecules. 2023;28(15):5931. doi:10.3390/molecules28155931; 37570901 PMC10421015

[14] Toews P, Bates J. Influence of drug and polymer molecular weight on release kinetics from HEMA and HPMA hydrogels. Sci Rep. 2023;13(1):16685. doi:10.1038/s41598-023-42923-3; 37794078 PMC10550905

[15] Siepmann J, Peppas NA. Modeling of drug release from delivery systems based on hydroxypropyl methylcellulose (HPMC). Adv Drug Deliv Rev. 2001;48(2–3):139–57. doi:10.1016/s0169-409x(01)00112-0; 11369079

[16] Li J, Mooney DJ. Designing hydrogels for controlled drug delivery. Nat Rev Mater. 2016;1:16071. doi:10.1038/natrevmats.2016.71; 29657852 PMC5898614

[17] Ávila-Salas F, Durán-Lara EF. An overview of injectable thermo-responsive hydrogels and advances in their biomedical applications. Curr Med Chem. 2020;27(34):5773–89. doi:10.2174/0929867325666190603110045; 31161984

[18] Siboro SAP, Anugrah DSB, Ramesh K, Park SH, Kim HR, Lim KT. Tunable porosity of covalently crosslinked alginate-based hydrogels and its significance in drug release behavior. Carbohydr Polym. 2021;260:117779. doi:10.1016/j.carbpol.2021.117779; 33712135

[19] Correa S, Grosskopf AK, Lopez Hernandez H, Chan D, Yu AC, Stapleton LM, et al. Translational applications of hydrogels. Chem Rev. 2021;121(18):11385–457. doi:10.1021/acs.chemrev.0c01177; 33938724 PMC8461619

[20] Andrade F, Roca-Melendres MM, Durán-Lara EF, Rafael D, Schwartz S Jr. Stimuli-responsive hydrogels for cancer treatment: the role of pH, light, ionic strength and magnetic field. Cancers. 2021;13(5):1164. doi:10.3390/cancers13051164; 33803133 PMC7963181

[21] Cao H, Duan Y, Lin Q, Yang Y, Gong Z, Zhong Y, et al. Dual-loaded, long-term sustained drug releasing and thixotropic hydrogel for localized chemotherapy of cancer. Biomater Sci. 2019;7(7):2975–85. doi:10.1039/c9bm00540d; 31106800

[22] Wang QQ, Tan C, Qin G, Yao SK. Promising clinical applications of hydrogels associated with precise cancer treatment: a review. Technol Cancer Res Treat. 2023;22:1–7. doi:10.1177/15330338221150322; 36604973 PMC9829993

[23] Asadi K, Samiraninezhad N, Akbarizadeh AR, Amini A, Gholami A. Stimuli-responsive hydrogel based on natural polymers for breast cancer. Front Chem. 2024;12:1325204. doi:10.3389/fchem.2024.1325204; 38304867 PMC10830687

[24] Hajareh Haghighi F, Binaymotlagh R, Fratoddi I, Chronopoulou L, Palocci C. Peptide-hydrogel nanocomposites for anti-cancer drug delivery. Gels. 2023;9(12):953. doi:10.3390/gels9120953; 38131939 PMC10742474

[25] Liscano Y, Oñate-Garzón J, Delgado JP. Peptides with dual antimicrobial-anticancer activity: strategies to overcome peptide limitations and rational design of anticancer peptides. Molecules. 2020;25(18):4245. doi:10.3390/molecules25184245; 32947811 PMC7570524

[26] Ghaly G, Tallima H, Dabbish E, Badr ElDin N, Abd El-Rahman MK, Ibrahim MAA, et al. Anti-cancer peptides: status and future prospects. Molecules. 2023;28(3):1148. doi:10.3390/molecules28031148; 36770815 PMC9920184

[27] Zhang QY, Yan ZB, Meng YM, Hong XY, Shao G, Ma JJ, et al. Antimicrobial peptides: mechanism of action, activity and clinical potential. Mil Med Res. 2021;8(1):48. doi:10.1186/s40779-021-00343-2; 34496967 PMC8425997

[28] Kordi M, Borzouyi Z, Chitsaz S, Asmaei MH, Salami R, Tabarzad M. Antimicrobial peptides with anticancer activity: today status, trends and their computational design. Arch Biochem Biophys. 2023;733:109484. doi:10.1016/j.abb.2022.109484; 36473507

[29] Resina L, Esteves T, Pérez-Rafael S, García JIH, Ferreira FC, Tzanov T, et al. Dual electro-/ pH-responsive nanoparticle/hydrogel system for controlled delivery of anticancer peptide. Biomater Adv. 2024;162:213925. doi:10.1016/j.bioadv.2024.213925; 38908101

[30] Chen C, Zhang Y, Hou Z, Cui X, Zhao Y, Xu H. Rational design of short peptide-based hydrogels with MMP-2 responsiveness for controlled anticancer peptide delivery. Biomacromolecules. 2017;18(11):3563–71. doi:10.1021/acs.biomac.7b00911; 28828862

[31] Yang L, Zhang C, Ren C, Liu J, Zhang Y, Wang J, et al. Supramolecular hydrogel based on chlorambucil and peptide drug for cancer combination therapy. ACS Appl Mater Interfaces. 2019;11(1):331–9. doi:10.1021/acsami.8b18425; 30560665

[32] Liu N, Wu S, Tian X, Li X. Fabrication of injectable hydrogels from an anticancer peptide for local therapeutic delivery and synergistic photothermal-chemotherapy. J Mater Chem B. 2022;10(27):5165–73. doi:10.1039/d2tb00917j; 35734944

[33] Castelletto V, Edwards-Gayle CJC, Greco F, Hamley IW, Seitsonen J, Ruokolainen J. Self-assembly, tunable hydrogel properties, and selective anti-cancer activity of a carnosine-derived lipidated peptide. ACS Appl Mater Interfaces. 2019;11(37):33573–80. doi:10.1021/acsami.9b09065; 31407889 PMC7007010

[34] Feng JP, Zhu R, Jiang F, Xie J, Gao C, Li M, et al. Melittin-encapsulating peptide hydrogels for enhanced delivery of impermeable anticancer peptides. Biomater Sci. 2020;8(16):4559–69. doi:10.1039/c9bm02080b; 32672773

[35] Ren C, Gao Y, Liu J, Zhang Y, Pu G, Yang L, et al. Anticancer supramolecular hydrogel of D/L-peptide with enhanced stability and bioactivity. J Biomed Nanotechnol. 2018;14(6):1125–34. doi:10.1166/jbn.2018.2564; 29843877

[36] Al Ojaimi Y, Blin T, Lamamy J, Gracia M, Pitiot A, Denevault-Sabourin C, et al. Therapeutic antibodies–natural and pathological barriers and strategies to overcome them. Pharmacol Ther. 2022;233:108022. doi:10.1016/j.pharmthera.2021.108022; 34687769 PMC8527648

[37] Chen X, Wang M, Yang X, Wang Y, Yu L, Sun J, et al. Injectable hydrogels for the sustained delivery of a HER2-targeted antibody for preventing local relapse of HER2+ breast cancer after breast-conserving surgery. Theranostics. 2019;9(21):6080–98. doi:10.7150/thno.36514; 31534538 PMC6735507

[38] Salehi-Had S, Saltzman WM. Controlled intracranial delivery of antibodies in the rat. In: Cleland JL, Ro L, editors. Formulation and delivery of proteins and peptides. Washington, DC, USA: American Chemical Society; 1994. p. 278–91. doi:10.1021/bk-1994-0567.ch016.

[39] Parkhurst MR, Saltzman WM. Controlled delivery of antibodies against leukocyte adhesion molecules from polymer matrices. J Control Release. 1996;42(3):273–88. doi:10.1016/0168-3659(96)01464-2.

[40] Awwad S, Abubakre A, Angkawinitwong U, Khaw PT, Brocchini S. *In situ* antibody-loaded hydrogel for intravitreal delivery. Eur J Pharm Sci. 2019;137:104993. doi:10.1016/j.ejps.2019.104993; 31302214

[41] Couzin-Frankel J. Cancer immunotherapy. Science. 2013;342(6165):1432–3. doi:10.1126/science.342.6165.1432; 24357284

[42] Waldman AD, Fritz JM, Lenardo MJ. A guide to cancer immunotherapy: from T cell basic science to clinical practice. Nat Rev Immunol. 2020;20(11):651–68. doi:10.1038/s41577-020-0306-5; 32433532 PMC7238960

[43] Zhang X, Guo X, Wu Y, Gao J. Locally injectable hydrogels for tumor immunotherapy. Gels. 2021;7(4):224. doi:10.3390/gels7040224; 34842684 PMC8628785

[44] Erfani A, Diaz AE, Doyle PS. Hydrogel-enabled, local administration and combinatorial delivery of immunotherapies for cancer treatment. Mater Today. 2023;65:227–43. doi:10.1016/j.mattod.2023.03.006.

[45] Zheng H, Li M, Wu L, Liu W, Liu Y, Gao J, et al. Progress in the application of hydrogels in immunotherapy of gastrointestinal tumors. Drug Deliv. 2023;30(1):2161670. doi:10.1080/10717544.2022.2161670; 36587630 PMC9809389

[46] Chen M, Tan Y, Dong Z, Lu J, Han X, Jin Q, et al. Injectable anti-inflammatory nanofiber hydrogel to achieve systemic immunotherapy post local administration. Nano Lett. 2020;20(9):6763–73. doi:10.1021/acs.nanolett.0c02684; 32787149

[47] Chohan KL, Siegler EL, Kenderian SS. CAR-T cell therapy: the efficacy and toxicity balance. Curr Hematol Malig Rep. 2023;18(2):9–18. doi:10.1007/s11899-023-00687-7; 36763238 PMC10505056

[48] Hu Q, Li H, Archibong E, Chen Q, Ruan H, Ahn S, et al. Inhibition of post-surgery tumour recurrence via a hydrogel releasing CAR-T cells and anti-PDL1-conjugated platelets. Nat Biomed Eng. 2021;5(9):1038–47. doi:10.1038/s41551-021-00712-1; 33903744 PMC9102991

[49] Lin MJ, Svensson-Arvelund J, Lubitz GS, Marabelle A, Melero I, Brown BD, et al. Cancer vaccines: the next immunotherapy frontier. Nat Cancer. 2022;3(8):911–26. doi:10.1038/s43018-022-00418-6; 35999309

[50] Yang A, Bai Y, Dong X, Ma T, Zhu D, Mei L, et al. Hydrogel/nanoadjuvant-mediated combined cell vaccines for cancer immunotherapy. Acta Biomater. 2021;133:257–67. doi:10.1016/j.actbio.2021.08.014; 34407475

[51] Yang F, Shi K, Jia Y, Hao Y, Peng J, Yuan L, et al. A biodegradable thermosensitive hydrogel vaccine for cancer immunotherapy. Appl Mater Today. 2020;19:100608. doi:10.1016/j.apmt.2020.100608.

[52] Yang P, Song H, Qin Y, Huang P, Zhang C, Kong D, et al. Engineering dendritic-cell-based vaccines and PD-1 blockade in self-assembled peptide nanofibrous hydrogel to amplify antitumor T-cell immunity. Nano Lett. 2018;18(7):4377–85. doi:10.1021/acs.nanolett.8b01406; 29932335

[53] Principe DR, Kamath SD, Korc M, Munshi HG. The immune modifying effects of chemotherapy and advances in chemo-immunotherapy. Pharmacol Ther. 2022;236:108111. doi:10.1016/j.pharmthera.2022.108111; 35016920 PMC9271143

[54] Wang F, Xu D, Su H, Zhang W, Sun X, Monroe MK, et al. Supramolecular prodrug hydrogelator as an immune booster for checkpoint blocker-based immunotherapy. Sci Adv. 2020;6(18):eaaz8985. doi:10.1126/sciadv.aaz8985; 32490201 PMC7239700

[55] Ashrafizadeh M, Farhood B, Eleojo Musa A, Taeb S, Rezaeyan A, Najafi M. Abscopal effect in radioimmunotherapy. Int Immunopharmacol. 2020;85:106663. doi:10.1016/j.intimp.2020.106663; 32521494

[56] Zaheer J, Kim H, Lee YJ, Kim JS, Lim SM. Combination radioimmunotherapy strategies for solid tumors. Int J Mol Sci. 2019;20(22):5579. doi:10.3390/ijms20225579; 31717302 PMC6888084

[57] Zhang Y, Feng Z, Liu J, Li H, Su Q, Zhang J, et al. Polarization of tumor-associated macrophages by TLR7/8 conjugated radiosensitive peptide hydrogel for overcoming tumor radioresistance. Bioact Mater. 2022;16:359–71. doi:10.1016/j.bioactmat.2021.12.033; 35386314 PMC8965723

[58] Assistance Publique—Hôpitaux de Paris. Local immunomodulation after radiofrequency of unresectable colorectal liver metastases (LICoRN-01). NCT04062721. [cited 2025 Jan 1]. Available from: https://classic.clinicaltrials.gov/ct2/show/NCT04062721.

[59] Xu P, Liang F. Nanomaterial-based tumor photothermal immunotherapy. Int J Nanomed. 2020;15:9159–80. doi:10.2147/IJN.S249252; 33244232 PMC7684030

[60] Li X, Lovell JF, Yoon J, Chen X. Clinical development and potential of photothermal and photodynamic therapies for cancer. Nat Rev Clin Oncol. 2020;17(11):657–74. doi:10.1038/s41571-020-0410-2; 32699309

[61] Jia YP, Shi K, Yang F, Liao JF, Han RX, Yuan LP, et al. Multifunctional nanoparticle loaded injectable thermoresponsive hydrogel as NIR controlled release platform for local photothermal immunotherapy to prevent breast cancer postoperative recurrence and metastases. Adv Funct Mater. 2020;30(25):2001059. doi:10.1002/adfm.202001059.

[62] Tang R, Xu Z. Gene therapy: a double-edged sword with great powers. Mol Cell Biochem. 2020;474(1–2):73–81. doi:10.1007/s11010-020-03834-3; 32696132

[63] Yahya EB, Alqadhi AM. Recent trends in cancer therapy: a review on the current state of gene delivery. Life Sci. 2021;269:119087. doi:10.1016/j.lfs.2021.119087; 33476633

[64] Martínez-Puente DH, Pérez-Trujillo JJ, Zavala-Flores LM, García-García A, Villanueva-Olivo A, Rodríguez-Rocha H, et al. Plasmid DNA for therapeutic applications in cancer. Pharmaceutics. 2022;14(9):1861. doi:10.3390/pharmaceutics14091861; 36145609 PMC9503848

[65] Zhang H, Huang JJ, Wang J, Hu M, Chen XC, Sun W, et al. Surface-mediated stimuli-responsive gene delivery based on breath figure film combined with matrix metalloproteinase-sensitive hydrogel. ACS Biomater Sci Eng. 2019;5(12):6610–6. doi:10.1021/acsbiomaterials.9b01353; 33423480

[66] Sioud M. RNA interference: story and mechanisms. Methods Mol Biol. 2021;2282:1–15. doi:10.1007/978-1-0716-1298-9_1; 33928566

[67] Lu YJ, Lan YH, Chuang CC, Lu WT, Chan LY, Hsu PW, et al. Injectable thermo-sensitive chitosan hydrogel containing CPT-11-loaded EGFR-targeted graphene oxide and SLP2 shRNA for localized drug/gene delivery in glioblastoma therapy. Int J Mol Sci. 2020;21(19):7111. doi:10.3390/ijms21197111; 32993166 PMC7583917

[68] Chen M, Mao A, Xu M, Weng Q, Mao J, Ji J. CRISPR-Cas9 for cancer therapy: opportunities and challenges. Cancer Lett. 2019;447:48–55. doi:10.1016/j.canlet.2019.01.017; 30684591

[69] Chen Z, Liu F, Chen Y, Liu J, Wang X, Chen AT, et al. Targeted delivery of CRISPR/Cas9-mediated cancer gene therapy via liposome-templated hydrogel nanoparticles. Adv Funct Mater. 2017;27(46):1703036. doi:10.1002/adfm.201703036; 29755309 PMC5939593

[70] Mondal M, Guo J, He P, Zhou D. Recent advances of oncolytic virus in cancer therapy. Hum Vaccin Immunother. 2020;16(10):2389–402. doi:10.1080/21645515.2020.1723363; 32078405 PMC7644205

[71] Du YN, Wei Q, Zhao LJ, Fan CQ, Guo LR, Ye JF, et al. Hydrogel-based co-delivery of CIK cells and oncolytic adenovirus armed with IL12 and IL15 for cancer immunotherapy. Biomed Pharmacother. 2022;151:113110. doi:10.1016/j.biopha.2022.113110; 35605298

[72] Yang C, Lee A, Gao S, Liu S, Hedrick JL, Yang YY. Hydrogels with prolonged release of therapeutic antibody: block junction chemistry modification of ‘ABA’ copolymers provides superior anticancer efficacy. J Control Release. 2019;293:193–200. doi:10.1016/j.jconrel.2018.11.026; 30521830

[73] Firouzabadi BM, Gigliobianco MR, Agas D, Sabbieti MG, Alimenti C, Devi LS, et al. Stimuli-sensitive hyaluronic acid hydrogels for localized and controlled release of antibodies. Eur J Pharm Biopharm. 2025;214:114804. doi:10.1016/j.ejpb.2025.114804; 40639449

[74] Lo YW, Sheu MT, Chiang WH, Chiu YL, Tu CM, Wang WY, et al. *In situ* chemically crosslinked injectable hydrogels for the subcutaneous delivery of trastuzumab to treat breast cancer. Acta Biomater. 2019;86(1):280–90. doi:10.1016/j.actbio.2019.01.003; 30616077

[75] Chung CK, Fransen MF, van der Maaden K, Campos Y, García-Couce J, Kralisch D, et al. Thermosensitive hydrogels as sustained drug delivery system for CTLA-4 checkpoint blocking antibodies. J Control Release. 2020;323:1–11. doi:10.1016/j.jconrel.2020.03.050; 32247805

[76] Kim J, Francis DM, Thomas SN. *In situ* crosslinked hydrogel depot for sustained antibody release improves immune checkpoint blockade cancer immunotherapy. Nanomaterials. 2021;11(2):471. doi:10.3390/nano11020471; 33673289 PMC7918828

[77] Grosskopf AK, Labanieh L, Klysz DD, Roth GA, Xu P, Adebowale O, et al. Delivery of CAR-T cells in a transient injectable stimulatory hydrogel niche improves treatment of solid tumors. Sci Adv. 2022;8(14):eabn8264. doi:10.1126/sciadv.abn8264; 35394838 PMC8993118

[78] Wang F, Huang Q, Su H, Sun M, Wang Z, Chen Z, et al. Self-assembling paclitaxel-mediated stimulation of tumor-associated macrophages for postoperative treatment of glioblastoma. Proc Natl Acad Sci U S A. 2023;120(18):e2204621120. doi:10.1073/pnas.2204621120; 37098055 PMC10161130

[79] Liu M, Cao Z, Zhang R, Chen Y, Yang X. Injectable supramolecular hydrogel for locoregional immune checkpoint blockade and enhanced cancer chemo-immunotherapy. ACS Appl Mater Interfaces. 2021;13(29):33874–84. doi:10.1021/acsami.1c08285; 34275267

[80] Lemdani K, Seguin J, Lesieur C, Al Sabbagh C, Doan BT, Richard C, et al. Mucoadhesive thermosensitive hydrogel for the intra-tumoral delivery of immunomodulatory agents, *in vivo* evidence of adhesion by means of non-invasive imaging techniques. Int J Pharm. 2019;567:118421. doi:10.1016/j.ijpharm.2019.06.012; 31176849

[81] Dong X, Yang A, Bai Y, Kong D, Lv F. Dual fluorescence imaging-guided programmed delivery of doxorubicin and CpG nanoparticles to modulate tumor microenvironment for effective chemo-immunotherapy. Biomaterials. 2020;230:119659. doi:10.1016/j.biomaterials.2019.119659; 31831223

[82] Kim J, Archer PA, Manspeaker MP, Avecilla ARC, Pollack BP, Thomas SN. Sustained release hydrogel for durable locoregional chemoimmunotherapy for BRAF-mutated melanoma. J Control Release. 2023;357:655–68. doi:10.1016/j.jconrel.2023.04.028; 37080489 PMC10328138

[83] Li J, Luo G, Zhang C, Long S, Guo L, Yang G, et al. *In situ* injectable hydrogel-loaded drugs induce anti-tumor immune responses in melanoma immunochemotherapy. Mater Today Bio. 2022;14:100238. doi:10.1016/j.mtbio.2022.100238; 35330634 PMC8938887

[84] Zhang H, Liu K, Gong Y, Zhu W, Zhu J, Pan F, et al. Vitamin C supramolecular hydrogel for enhanced cancer immunotherapy. Biomaterials. 2022;287:121673. doi:10.1016/j.biomaterials.2022.121673; 35839587

[85] Wang B, Chen J, Caserto JS, Wang X, Ma M. An *in situ* hydrogel-mediated chemo-immunometabolic cancer therapy. Nat Commun. 2022;13(1):3821. doi:10.1038/s41467-022-31579-8; 35780226 PMC9250515

[86] Jin HS, Choi DS, Ko M, Kim D, Lee DH, Lee S, et al. Extracellular pH modulating injectable gel for enhancing immune checkpoint inhibitor therapy. J Control Release. 2019;315(1):65–75. doi:10.1016/j.jconrel.2019.10.041; 31669264

[87] Gu J, Zhao G, Yu J, Xu P, Yan J, Jin Z, et al. Injectable pH-responsive hydrogel for combinatorial chemoimmunotherapy tailored to the tumor microenvironment. J Nanobiotechnol. 2022;20(1):372. doi:10.1186/s12951-022-01561-z; 35953828 PMC9367026

[88] Li S, Zhu C, Zhou X, Chen L, Bo X, Shen Y, et al. Engineering ROS-responsive bioscaffolds for disrupting myeloid cell-driven immunosuppressive niche to enhance PD-L1 blockade-based postablative immunotherapy. Adv Sci. 2022;9(11):2104619. doi:10.1002/advs.202104619; 35156339 PMC9008797

[89] Liu Y, Han YY, Lu S, Wu Y, Li J, Sun X, et al. Injectable hydrogel platform with biodegradable Dawson-type polyoxometalate and R848 for combinational photothermal-immunotherapy of cancer. Biomater Sci. 2022;10(5):1257–66. doi:10.1039/d1bm01835c; 35080214

[90] Fakhari A, Nugent S, Elvecrog J, Vasilakos J, Corcoran M, Tilahun A, et al. Thermosensitive gel–based formulation for intratumoral delivery of toll-like receptor 7/8 dual agonist, MEDI9197. J Pharm Sci. 2017;106(8):2037–45. doi:10.1016/j.xphs.2017.04.041; 28456734

[91] Liu Q, Zhang D, Qian H, Chu Y, Yang Y, Shao J, et al. Superior antitumor efficacy of IFN-α2b-incorporated photo-cross-linked hydrogels combined with T cell transfer and low-dose irradiation against gastric cancer. Int J Nanomed. 2020;15:3669–80. doi:10.2147/IJN.S249174; 32547021 PMC7261665

[92] Li C, Liu Y, Li D, Wang Q, Zhou S, Zhang H, et al. Promising alternatives of CD47 monoclonal antibody: an injectable degradable hydrogel loaded with PQ912 for postoperative immunotherapy effectively blocks CD47-SIRPα signal. Theranostics. 2022;12(10):4581–98. doi:10.7150/thno.72310; 35832081 PMC9254232

[93] Li S, Zhang W, Xing R, Yuan C, Xue H, Yan X. Supramolecular nanofibrils formed by coassembly of clinically approved drugs for tumor photothermal immunotherapy. Adv Mater. 2021;33(29):e2103733. doi:10.1002/adma.202103733; 33876464

[94] Zhang Y, Wang T, Zhuang Y, He T, Wu X, Su L, et al. Sodium alginate hydrogel-mediated cancer immunotherapy for postoperative *in situ* recurrence and metastasis. ACS Biomater Sci Eng. 2021;7(12):5717–26. doi:10.1021/acsbiomaterials.1c01216; 34757733

[95] Wu Y, Li Q, Shim G, Oh YK. Melanin-loaded CpG DNA hydrogel for modulation of tumor immune microenvironment. J Control Release. 2021;330(1):540–53. doi:10.1016/j.jconrel.2020.12.040; 33373649

[96] Yao C, Zhu C, Tang J, Ou J, Zhang R, Yang D. T lymphocyte-captured DNA network for localized immunotherapy. J Am Chem Soc. 2021;143(46):19330–40. doi:10.1021/jacs.1c07036; 34780151

[97] Yin Y, Li X, Ma H, Zhang J, Yu D, Zhao R, et al. *In situ* transforming RNA nanovaccines from polyethylenimine functionalized graphene oxide hydrogel for durable cancer immunotherapy. Nano Lett. 2021;21(5):2224–31. doi:10.1021/acs.nanolett.0c05039; 33594887

[98] Le TMD, Jung BK, Li Y, Duong HTT, Nguyen TL, Hong JW, et al. Physically crosslinked injectable hydrogels for long-term delivery of oncolytic adenoviruses for cancer treatment. Biomater Sci. 2019;7(10):4195–207. doi:10.1039/c9bm00992b; 31386700

[99] Duong HTT, Thambi T, Yin Y, Kim SH, Nguyen TL, Giang Phan VH, et al. Degradation-regulated architecture of injectable smart hydrogels enhances humoral immune response and potentiates antitumor activity in human lung carcinoma. Biomaterials. 2020;230:119599. doi:10.1016/j.biomaterials.2019.119599; 31718883

[100] Chalanqui MJ, Pentlavalli S, McCrudden C, Chambers P, Ziminska M, Dunne N, et al. Influence of alginate backbone on efficacy of thermo-responsive alginate-g-P(NIPAAm) hydrogel as a vehicle for sustained and controlled gene delivery. Mater Sci Eng C Mater Biol Appl. 2019;95:409–21. doi:10.1016/j.msec.2017.09.003; 30573265

[101] Zhao D, Song H, Zhou X, Chen Y, Liu Q, Gao X, et al. Novel facile thermosensitive hydrogel as sustained and controllable gene release vehicle for breast cancer treatment. Eur J Pharm Sci. 2019;134:145–52. doi:10.1016/j.ejps.2019.03.021; 30926401

[102] Ding L, Li J, Wu C, Yan F, Li X, Zhang S. A self-assembled RNA-triple helix hydrogel drug delivery system targeting triple-negative breast cancer. J Mater Chem B. 2020;8(16):3527–33. doi:10.1039/c9tb01610d; 31737891

[103] Słyk Ż, Wrzesień R, Barszcz S, Gawrychowski K, Małecki M. Adeno-associated virus vector hydrogel formulations for brain cancer gene therapy applications. Biomed Pharmacother. 2024;170:116061. doi:10.1016/j.biopha.2023.116061; 38154269

[104] Parvin N, Joo SW, Mandal TK. Injectable biopolymer-based hydrogels: a next-generation platform for minimally invasive therapeutics. Gels. 2025;11(6):383. doi:10.3390/gels11060383; 40558682 PMC12192118

[105] Baweja R, Ravi R, Baweja R, Gupta S, Sachan A, Pratyusha V, et al. A comprehensive review of hydrogels as potential drug carriers for anticancer therapies: properties, development and future prospects. Next Mater. 2025;8:100913. doi:10.1016/j.nxmate.2025.100913.

[106] Liu Z, Ma X, Liu J, Zhang H, Fu D. Advances in the application of natural/synthetic hybrid hydrogels in tissue engineering and delivery systems: a comprehensive review. Int J Pharm. 2025;672:125323. doi:10.1016/j.ijpharm.2025.125323; 39923883

[107] Rana MM, de la Hoz Siegler H. Evolution of hybrid hydrogels: next-generation biomaterials for drug delivery and tissue engineering. Gels. 2024;10(4):216. doi:10.3390/gels10040216; 38667635 PMC11049329

[108] Zhong R, Talebian S, Mendes BB, Wallace G, Langer R, Conde J, et al. Hydrogels for RNA delivery. Nat Mater. 2023;22(7):818–31. doi:10.1038/s41563-023-01472-w; 36941391 PMC10330049

[109] Nassar N, Kasapis S. Fundamental advances in hydrogels for the development of the next generation of smart delivery systems as biopharmaceuticals. Int J Pharm. 2023;633:122634. doi:10.1016/j.ijpharm.2023.122634; 36690133

[110] Picchioni F, Muljana H, Picchioni F, Muljana H. Hydrogels based on dynamic covalent and non covalent bonds: a chemistry perspective. Gels. 2018;4(1):21. doi:10.3390/gels4010021; 30674797 PMC6318606

[111] Andrade F, Roca-Melendres MM, Llaguno M, Hide D, Raurell I, Martell M, et al. Smart and eco-friendly N-isopropylacrylamide and cellulose hydrogels as a safe dual-drug local cancer therapy approach. Carbohydr Polym. 2022;295:119859. doi:10.1016/j.carbpol.2022.119859; 35988981

[112] Yoon MS, Lee JM, Jo MJ, Kang SJ, Yoo MK, Park SY, et al. Dual-drug delivery systems using hydrogel-nanoparticle composites: recent advances and key applications. Gels. 2025;11(7):520. doi:10.3390/gels11070520; 40710682 PMC12294678

[113] Vermonden T, Censi R, Hennink WE. Hydrogels for protein delivery. Chem Rev. 2012;112(5):2853–88. doi:10.1021/cr200157d; 22360637

[114] Segneanu AE, Bejenaru LE, Bejenaru C, Blendea A, Mogoşanu GD, Biţă A, et al. Advancements in hydrogels: a comprehensive review of natural and synthetic innovations for biomedical applications. Polymers. 2025;17(15):2026. doi:10.3390/polym17152026; 40808075 PMC12349326

[115] Lu P, Ruan D, Huang M, Tian M, Zhu K, Gan Z, et al. Harnessing the potential of hydrogels for advanced therapeutic applications: current achievements and future directions. Signal Transduct Target Ther. 2024;9(1):166. doi:10.1038/s41392-024-01852-x; 38945949 PMC11214942

[116] Fernandez-Yague MA, Hymel LA, Olingy CE, McClain C, Ogle ME, García JR, et al. Analyzing immune response to engineered hydrogels by hierarchical clustering of inflammatory cell subsets. Sci Adv. 2022;8(8):eabd8056. doi:10.1126/sciadv.abd8056; 35213226 PMC8880784

[117] Choi H, Choi WS, Jeong JO. A review of advanced hydrogel applications for tissue engineering and drug delivery systems as biomaterials. Gels. 2024;10(11):693. doi:10.3390/gels10110693; 39590049 PMC11594258

[118] Lanis MR, Kim S, Schneck JP. Hydrogels in the immune context: *in vivo* applications for modulating immune responses in cancer therapy. Gels. 2025;11(11):889. doi:10.3390/gels11110889; 41294574 PMC12652042

[119] Wu Y, Xiao Y, Yin B, Wong SHD. Dynamic hydrogels: adaptive biomaterials for engineering tumor microenvironment and cancer treatment. Int J Mol Sci. 2025;26(19):9502. doi:10.3390/ijms26199502; 41096764 PMC12525343

